# Clinical and prognostic associations of autoantibodies recognizing adrenergic/muscarinic receptors in patients with heart failure

**DOI:** 10.1093/cvr/cvad042

**Published:** 2023-03-08

**Authors:** George Markousis-Mavrogenis, Waldemar B Minich, Ali A Al-Mubarak, Stefan D Anker, John G F Cleland, Kenneth Dickstein, Chim C Lang, Leong L Ng, Nilesh J Samani, Faiez Zannad, Marco Metra, Petra Seemann, Antonia Hoeg, Patricio Lopez, Dirk J van Veldhuisen, Rudolf A de Boer, Adriaan A Voors, Peter van der Meer, Lutz Schomburg, Nils Bomer, The BIOSTAT-CHF Consortium

**Affiliations:** Department of Cardiology, University Medical Center Groningen, University of Groningen, Hanzeplein 1, 9713 GZ Groningen, The Netherlands; Institute for Experimental Endocrinology, Charité-Universitätsmedizin Berlin, Hessische Straß0065 4A, CCM, Berlin D-10115, Germany; ImmunometriX GmbH i.L, Brandenburgische Str. 83, D-10713 Berlin, Germany; Department of Cardiology, University Medical Center Groningen, University of Groningen, Hanzeplein 1, 9713 GZ Groningen, The Netherlands; Department of Cardiology (CVK) of German Heart Center Charité; Institute of Health Center for Regenerative Therapies (BCRT), German Centre for Cardiovascular Research (DZHK) partner site Berlin, Charité Universitätsmedizin, Charitépl. 1, 10117 Berlin, Germany; Institute of Cardiovascular and Medical Sciences, University of Glasgow, University Avenue, Glasgow G12 8QQ, UK; National Heart & Lung Institute, Imperial College, Guy Scadding Building, Dovehouse St, London SW3 6LY, UK; University of Bergen, Stavanger University Hospital, Gerd-Ragna Bloch Thorsens gate 8, 4011 Stavanger, Norway; Division of Molecular & Clinical Medicine, University of Dundee, Nethergate, Dundee DD1 4HN, UK; Department of Cardiovascular Sciences, University of Leicester, Glenfield Hospital, Groby Rd, Leicester LE3 9QP, UK; NIHR Leicester Biomedical Research Centre, Glenfield Hospital, Groby Rd, Leicester LE3 9QP, UK; University of Bergen, Stavanger University Hospital, Gerd-Ragna Bloch Thorsens gate 8, 4011 Stavanger, Norway; Université de Lorraine, Inserm CIC 1403, CHRU, Cité Universitaire, 57000 Metz, France; Cardiology, ASST Spedali Civili, Department of Medical and Surgical Specialties, Radiological Sciences and Public Health, University of Brescia, Piazza del Mercato, 15, 25121 Brescia BS, Italy; Institute for Experimental Endocrinology, Charité-Universitätsmedizin Berlin, Hessische Straß0065 4A, CCM, Berlin D-10115, Germany; ImmunometriX GmbH i.L, Brandenburgische Str. 83, D-10713 Berlin, Germany; Institute for Experimental Endocrinology, Charité-Universitätsmedizin Berlin, Hessische Straß0065 4A, CCM, Berlin D-10115, Germany; Institute for Experimental Endocrinology, Charité-Universitätsmedizin Berlin, Hessische Straß0065 4A, CCM, Berlin D-10115, Germany; ImmunometriX GmbH i.L, Brandenburgische Str. 83, D-10713 Berlin, Germany; Department of Cardiology, University Medical Center Groningen, University of Groningen, Hanzeplein 1, 9713 GZ Groningen, The Netherlands; Department of Cardiology, University Medical Center Groningen, University of Groningen, Hanzeplein 1, 9713 GZ Groningen, The Netherlands; Department of Cardiology, University Medical Center Groningen, University of Groningen, Hanzeplein 1, 9713 GZ Groningen, The Netherlands; Department of Cardiology, University Medical Center Groningen, University of Groningen, Hanzeplein 1, 9713 GZ Groningen, The Netherlands; Institute for Experimental Endocrinology, Charité-Universitätsmedizin Berlin, Hessische Straß0065 4A, CCM, Berlin D-10115, Germany; Department of Cardiology, University Medical Center Groningen, University of Groningen, Hanzeplein 1, 9713 GZ Groningen, The Netherlands

**Keywords:** Beta 1, Beta 2, Beta 3, M2, Immune system, Autoimmunity

## Abstract

**Aims:**

The importance of autoantibodies (AABs) against adrenergic/muscarinic receptors in heart failure (HF) is not well-understood. We investigated the prevalence and clinical/prognostic associations of four AABs recognizing the M2-muscarinic receptor or the β1-, β2-, or β3-adrenergic receptor in a large and well-characterized cohort of patients with HF.

**Methods and results:**

Serum samples from 2256 patients with HF from the BIOSTAT-CHF cohort and 299 healthy controls were analysed using newly established chemiluminescence immunoassays. The primary outcome was a composite of all-cause mortality and HF rehospitalization at 2-year follow-up, and each outcome was also separately investigated. Collectively, 382 (16.9%) patients and 37 (12.4%) controls were seropositive for ≥1 AAB (*P* = 0.045). Seropositivity occurred more frequently only for anti-M2 AABs (*P* = 0.025). Amongst patients with HF, seropositivity was associated with the presence of comorbidities (renal disease, chronic obstructive pulmonary disease, stroke, and atrial fibrillation) and with medication use. Only anti-β1 AAB seropositivity was associated with the primary outcome [hazard ratio (95% confidence interval): 1.37 (1.04–1.81), *P* = 0.024] and HF rehospitalization [1.57 (1.13–2.19), *P* = 0.010] in univariable analyses but remained associated only with HF rehospitalization after multivariable adjustment for the BIOSTAT-CHF risk model [1.47 (1.05–2.07), *P* = 0.030]. Principal component analyses showed considerable overlap in B-lymphocyte activity between seropositive and seronegative patients, based on 31 circulating biomarkers related to B-lymphocyte function.

**Conclusions:**

AAB seropositivity was not strongly associated with adverse outcomes in HF and was mostly related to the presence of comorbidities and medication use. Only anti-β1 AABs were independently associated with HF rehospitalization. The exact clinical value of AABs remains to be elucidated.

## Graphical Abstract

**Graphical Abstract:**
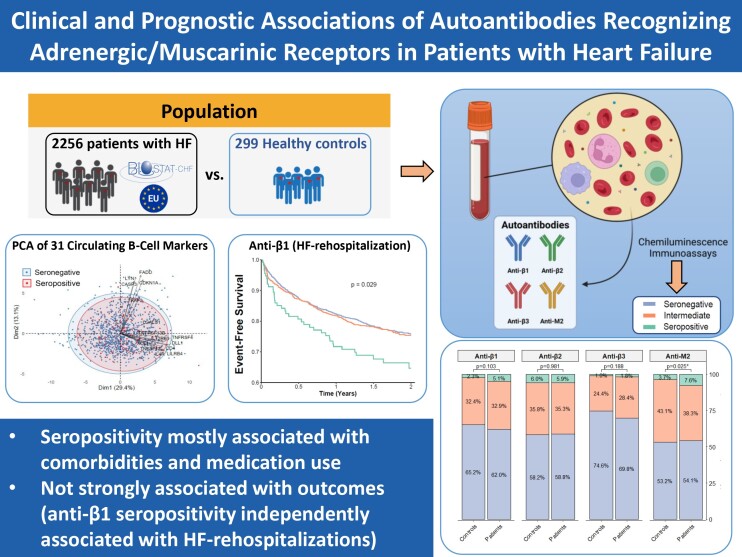

**Time of primary review: 38 days**

## 1. Introduction

Many studies have demonstrated the intricate relationship between immune activation and pathophysiological mechanisms in heart failure (HF).^1^ Nevertheless, direct immunomodulatory interventions have as of yet no established role in the clinical management of HF.

Antibodies are soluble forms of the B-cell receptor that are secreted by activated plasma cells and constitute an important part of the adaptive immune response.^2^ The generation of an effective immune response requires a broad antibody repertoire, which is achieved with impressive efficiency by recombination of immunoglobulin genes.^3^ This permits the generation of antibodies with potentially infinite specificities. Unavoidably, this process also leads to the generation of antibodies against self-antigens. Specialized regulatory mechanisms have evolved to eliminate self-reactive lymphocytes;^4^ nevertheless, these do not always function optimally. Accordingly, self-reactive lymphocytes and autoantibodies (AABs) feature prominently in numerous autoimmune rheumatic diseases^5^ and other disease states.^6^

A relationship between AABs against autonomic nervous system receptors (ANS-AABs) and HF has been described previously, particularly in patients with non-hereditary dilated cardiomyopathy (DCM)^7^ and Chagas cardiomyopathy.^8^ However, to our knowledge, most reports are limited to small cohorts, and no data from large, well-characterized, and diverse populations currently exist in the literature. We thus aimed to investigate the prevalence of ANS-AAB seropositivity in patients with HF and to determine its clinical associations and potential relationships with outcomes.

## 2. Methods

### 2.1 Patients

We performed a post hoc analysis of the BIOSTAT-CHF index cohort (*n* = 2516), which has been described previously.^9^ Briefly, BIOSTAT-CHF was a multi-centre study enrolling patients from 11 European countries. Participants were aged ≥18 years and had symptoms of new-onset or worsening HF, combined with a left ventricular ejection fraction (LVEF) ≤ 40% or brain-type natriuretic peptide (BNP) and/or *N*-terminal pro-BNP (NT-proBNP) plasma levels > 400 or >2000 pg/mL, respectively. Participants either were not previously treated with angiotensin-converting enzyme inhibitors/angiotensin receptor blockers (ACEi/ARB) and/or β-adrenoreceptor blockers (BB) or were receiving ≤50% of guideline-recommended target doses and anticipated their initiation or uptitration. All patients were treated with loop diuretics. Participants could be enrolled as inpatients or outpatients. The primary outcome was a composite of all-cause mortality and rehospitalization for HF censored at 2-year follow-up, with each component separately constituting a secondary outcome. The study protocol was approved by local and national ethics committees (EudraCT 2010-020808-29; R&D Ref Number 2008-CA03; MREC Number 10/S1402/39), and all participants provided written informed consent. The study was performed according to the principles outlined in the Declaration of Helsinki.

### 2.2 Autoantibody measurements

Measurements of AAB titres against β1-, β2-, and β3-adrenergic receptors, as well as M2-muscarinic receptors, were performed using chemiluminescence immunoassays (ImmunometriX, Berlin, Germany) on blood serum samples from 2256/2516 (89.7%) patients from the BIOSTAT-CHF index cohort and in 299 controls that self-identified as healthy [acquired commercially from in.vent Diagnostica (Berlin, Germany)]. Assay development and calculation of binding indices are described in the Supplementary material online, *Methods*. For quality control of the anti-β1 AAB assay, one negative sample and one positive sample from the control group (binding index below the median and higher than 10, respectively) were measured in duplicate in all microtitre plates during analysis (*n* = 26 duplicates each). As there is no prior knowledge on the threshold for relevant AAB concentrations, the 99th percentile value of negative controls (1.3883) was used to define a cut-off under which an assay would be considered definitively negative (henceforth ‘seronegative’), while the 1st percentile value of positive controls (7.3818) was similarly used to define a cut-off above which an assay would be considered definitively positive (henceforth ‘seropositive’) (see Supplementary material online, *Figure S1*). Patients with assay results in between the two cut-off values were labelled ‘intermediate’. The same cut-offs were also applied for defining subgroups in the remaining AAB assays.

### 2.3 Laboratory indices

The estimated glomerular filtration rate (eGFR) was calculated using the modification of diet in renal disease (MDRD) formula. NT-proBNP and hs-cTnT were measured using sandwich immunoassays (Roche Inc.), and CRP was measured using competitive immunoassays on a Luminex platform (Alere Inc.). Anaemia was defined according to the World Health Organization definition.

### 2.4 Biomarker measurements

Plasma biomarkers were determined using the proximity extension assay technology (Olink Proteomics Inc.), as part of four biomarker panels involving 92 biomarker measurements each (Cardiovascular-II, Cardiovascular-III, and Immune Response and Oncology II panels), thus 368 biomarkers in total. The complete list of available biomarkers has been reported previously.^10^ Overlapping biomarkers between the four panels were amphiregulin, c-kit ligand, and tissue factor pathway inhibitor-2 (measured twice) and interleukin-6 (IL-6) (measured three times). For overlapping biomarkers, the mean of all measurements was used, leaving a total of 363 unique biomarkers. Additionally, eight biomarkers were excluded from analyses because >10% of measurements were outside the assay’s limit of detection. Thus, in total, 355 biomarkers were available for further analysis.

### 2.5 Pathway over-representation analysis

Pathway over-representation analysis of the 355 biomarkers was performed using the ‘GProfiler’ pathway analyser (version e104_eg51_p15_3922dba).^11^ The results of the analysis were classified based on the gene ontology (GO) classification of biological processes (annotation 2022–01–13).^12^ Corrections for multiple testing were performed using the built-in g:SCS algorithm, using a false discovery rate threshold of 5%. Only significantly over-represented processes that included ≥5 of their constituents were included in the final selection. The biomarker corneodesmosin could not be analysed by GProfiler, and since the biomarkers BNP and NT-proBNP share the same protein designation, the effective number of analysed biomarkers was eventually 353.

### 2.6 Selection of GO biological processes related to B-lymphocytes and antibodies

**Figure 1:**
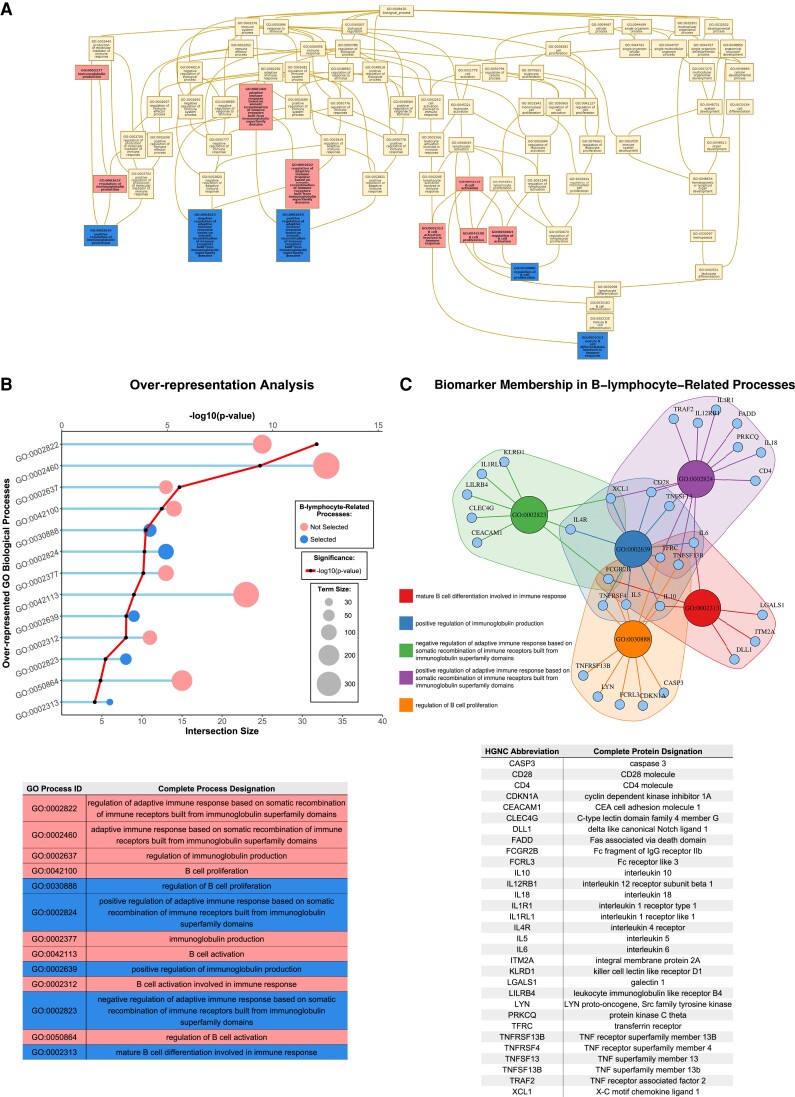
(*A*) Directed acyclic graph displaying the 13 over-represented GO biological processes containing the terms ‘B cell’ and ‘immunoglobulin’. Non-redundant children terms selected for further analysis are highlighted in blue. A higher resolution version of this image is presented in Supplementary material online, *Figure S2*. (*B*) Detailed results of pathway over-representation analysis for the 13 selected biological processes, including the *P*-value, intersection size, and term size. (*C*) Network denoting biomarker membership to the 5 GO biological processes that were used for further analyses. GO, gene ontology; ID, identifier; HGNC, HUGO Gene Nomenclature Committee.

The pathway over-representation analysis of the 353 biomarkers yielded 768 over-represented biological processes. Of these, 13 contained the terms ‘B cell’ and ‘immunoglobulin’. To eliminate potential data redundancies due to member overlap between processes,^10^ the 13 selected processes were visualized in a directed acyclic graph, and the most distal non-redundant processes were selected (*Figure 1A*, *Figure 1B*, Supplementary material online, *Figure S2*). This yielded five processes related to B-lymphocytes and antibodies, represented by different combinations of 31 biomarkers (*Figure 1C*).

### 2.7 Statistical analysis

Statistical analyses were conducted using R-studio (R version 4.2.0). Normality of continuous variables was determined by visual inspection of histograms and Q–Q plots. Baseline characteristics were compared for each of the four investigated AABs between seronegative, intermediate, and seropositive patients. Normally distributed variables were compared using one-way analysis of variance (ANOVA), non-normally distributed continuous variables using Kruskal–Wallis tests, and categorical/binary variables using chi-square tests. In case of a significant ANOVA result, pairwise post hoc testing with independent *t*-tests using Bonferroni correction was performed. Chemiluminescence assay results for each AAB were compared between patients with HF and healthy controls when considering the corresponding binding index as continuous variable using the Mann–Whitney *U* test and when classified based on the previously defined groups by using chi-square tests. Associations between AAB status and the primary outcome and all-cause mortality were investigated using Kaplan–Meier curves, while associations with hospitalization were investigated using cumulative incidence function curves while considering all-cause mortality a competing risk. Univariable associations were investigated with log-rank tests, while multivariable corrections for previously published risk models for this cohort^9^ were performed using Cox regression, when statistical assumptions were met. These included, amongst others, age, comorbidities, measures of congestion, NT-proBNP, and renal function.^9^ Statistical testing in survival analyses was performed using the log-rank test. Statistical significance was considered as *P* ≤ 0.05, considering the exploratory character of this study.

Significant findings from survival analyses were further verified with propensity score matching (MatchIt package v. 4.5.0) based on the aforementioned variables of the BIOSTAT-CHF risk model. Seropositive patients were matched 1 : 1 to seronegative patients. A nearest-neighbour matching algorithm with a caliper of 0.2 and without replacement, taking into account the region of common support, was used for this purpose.^13^ Unmatched observations were discarded. Statistical and graphical balance diagnostics tools were performed in order to evaluate the matching process.

To investigate the relative state of B-lymphocyte function in seronegative and seropositive patients, principal component analyses (PCA) were conducted for each AAB, by excluding patients that were categorized as intermediate. Subsequently, the results were plotted on biplots with concentration ellipses for each group.

## 3. Results

Baseline characteristics for the entire cohort are presented in *Table 1*. Mean age was 69 (12) years, and 602 (26.7%) were women. Ischaemia was the most prevalent primary aetiology of HF (46%), and median NT-proBNP was 2721.0 pg/mL (1204.3, 5741.8). Median LVEF was 30% (25–37), and 216 (10.7%) patients had an LVEF > 40%. Healthy controls had a median age of 32 years (24, 40) (range: 18–63 years) and 134 (44.8%) were women.

### 3.1 Comparison of assay-binding indices in healthy controls and patients with heart failure

**Figure 2:**
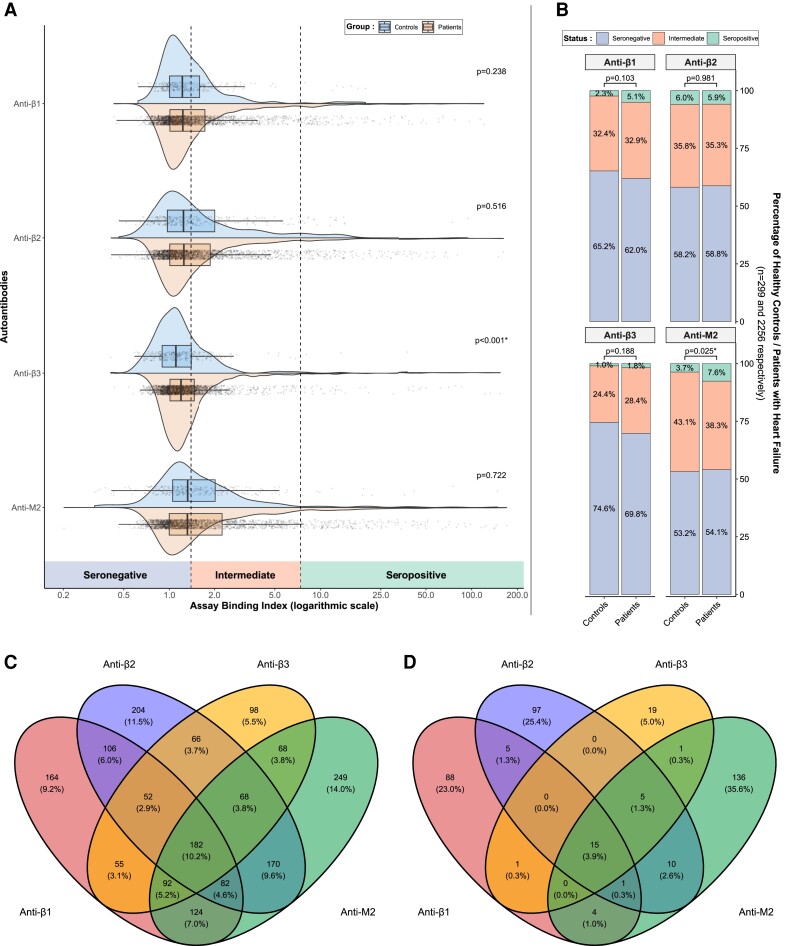
(*A*) Half-violin plots and boxplots with individual data points of assay-binding indices for all investigated autoantibodies, presented separately for healthy controls and patients with HF. (*B*) Relative prevalence of seronegative, intermediate, and seropositive status for each autoantibody in healthy controls and patients with HF. (*C*) Venn diagram illustrating the co-occurrence of intermediate and/or seropositive status for each autoantibody in patients with HF. (*D*) Venn diagram illustrating overlapping seropositivity for each autoantibody in patients with HF. HF, heart failure.

Assay-binding indices for each AAB are presented as continuous variables using half-violin plots with superimposed boxplots and individual data points in *Figure 2A*. There were no statistically significant differences between healthy controls and patients with HF in the median binding index for anti-β1 AABs [1.22 (1.00, 1.59) vs. 1.23 (1.01, 1.73), *P* = 0.238], anti-β2 AABs [1.24 (0.97, 2.01) vs. 1.25 (1.01, 1.88), *P* = 0.516], and anti-M2 AABs [1.32 (1.05, 2.02) vs. 1.31 (1.00, 2.25), *P* = 0.722]. Patients with HF had a higher median binding index for anti-β3 AABs [1.10 (0.89, 1.39) vs. 1.19 (1.01, 1.46), *P* < 0.001].

### 3.2 Classification of autoantibody status

The relative prevalence of seronegative, intermediate, and seropositive status amongst healthy controls and patients with HF is presented in *Figure 2B*. The prevalence of seropositivity amongst patients with HF in increasing order was 1.8% (*n* = 41) for anti-β3, 5.1% (*n* = 114) for anti-β1, 5.9% (*n* = 133) for anti-β2, and 7.6% (*n* = 172) for anti-M2 AABs. The proportion of groups was significantly different between patients with HF compared with healthy controls only for anti-M2 AABs, driven by a higher proportion of seropositive patients (*P* = 0.025). In total, 382 (16.9%) patients with HF were classified as seropositive for at least one AAB, compared with 37 (12.4%) of the healthy controls (*P* = 0.045). Summary statistics for assay-binding indices in total and stratified by AAB status are presented in Supplementary material online, *Tables S1* and *S2*, respectively.

The co-occurrence of either intermediate or seropositive status between the different AABs is presented in *Figure 2C*, while the co-occurrence of seropositive status only is presented in *Figure 2D*. Amongst the 382 patients that were seropositive for at least one AABs, co-occurrence of seropositivity for any of the remaining AABs had a low prevalence, with the highest value being observed for seropositivity for all four AABs in 15/382 (3.9%) patients. Collectively, 340/382 (89.0%) patients were seropositive for only one of the studied AABs.

### 3.3 Clinical associations of autoantibody seropositivity

Baseline characteristics stratified by seronegative, intermediate, and seropositive groupings for each AAB are presented in *Tables 1–4* for anti-β1, anti-β2, anti-β3, and anti-M2 AABs, respectively.

**Table 1:** Baseline characteristics for the entire patient cohort and stratified according to anti-β1-adrenergic receptor autoantibody status

Variable	Whole cohort	Anti-β1 seronegative	Anti-β1 intermediate	Anti-β1 seropositive	*P*-value
Group size	2256	1399	743	114	N/A
Demographics					
Age (years)	69 (12)	69 (12)	69 (11)	70 (12)	0.568
Female sex	602 (26.7%)	401 (28.7%)	167 (22.5%)	34 (29.8%)	**0**.006*
Recruited as outpatient	822 (32.6%)	455 (32.6%)	242 (32.6%)	37 (32.5%)	0.999
Primary HF aetiology					
Cardiomyopathy	556 (25.1%)	372 (27.1%)	169 (23.2%)	15 (13.4%)	**0**.002*
Hypertensive	233 (10.5%)	137 (10.0%)	85 (11.7%)	11 (9.8%)	0.468
Ischaemic	1018 (46.0%)	619 (45.1%)	342 (47.0%)	57 (50.9%)	0.402
Valvular disease	167 (7.5%)	104 (7.6%)	49 (6.7%)	14 (12.5%)	0.099
Previous history and comorbidities					
AF	1024 (45.4%)	636 (45.5%)	331 (44.5%)	57 (50.0%)	0.549
DM	732 (32.5%)	459 (32.9%)	237 (31.9%)	36 (31.6%)	0.884
HT	1398 (62.0%)	868 (62.1%)	458 (61.6%)	72 (63.2%)	0.944
RD	628 (27.9%)	366 (26.2%)	216 (29.1%)	46 (40.4%)	**0**.004*
Anaemia	746 (36.4%)	446 (35.1%)	250 (37.0%)	50 (47.2%)	**0**.043*
COPD	395 (17.5%)	232 (16.6%)	133 (17.9%)	30 (26.3%)	**0**.030*
MI	864 (38.3%)	525 (37.6%)	297 (40.0%)	42 (36.8%)	0.525
Stroke	212 (9.4%)	133 (9.5%)	70 (9.4%)	9 (7.9%)	0.849
HF hospitalization during previous year	711 (31.5%)	439 (31.4%)	242 (32.6%)	30 (26.3%)	0.404
Smoking					0.16
Never	824 (36.6%)	534 (38.3%)	252 (34.0%)	38 (33.6%)	
Past	1105 (49.1%)	656 (47.0%)	390 (52.6%)	59 (52.2%)	
Current	322 (14.3%)	206 (14.8%)	100 (13.5%)	16 (14.2%)	
Clinical characteristics					
NYHA functional class					
I	209 (10.6%)	132 (10.9%)	66 (10.1%)	11 (10.8%)	0.226
II	1014 (51.6%)	644 (53.2%)	323 (49.6%)	47 (46.1%)	
III	663 (33.8%)	391 (32.3%)	236 (36.3%)	36 (35.3%)	
IV	78 (4.0%)	44 (3.6%)	26 (4.0%)	8 (7.8%)	
NYHA class III or IV	741 (37.7%)	435 (35.9%)	262 (40.2%)	44 (43.1%)	0.095
BMI (kg/m^2^)	27.1 [24.1, 30.5]	27.2 [24.2, 30.8]	27.2 [24.0, 30.5]	25.9 [23.6, 29.1]	0.089
HR (b.p.m.)	76 [67, 90]	76 [67, 88]	76 [67, 90]	75 [67, 85]	0.813
SBP (mm Hg)	125 (22)	125 (22)	125 (23)	126 (21)	0.907
DBP (mm Hg)	75 (13)	75 (13)	75 (14)	73 (14)	0.179
Pulmonary crackles	1156 (52.8%)	716 (52.7%)	381 (53.1%)	59 (53.2%)	0.984
6MWT successfully completed	1413 (64.8%)	888 (65.7%)	459 (64.2%)	66 (58.4%)	0.266
6MWT distance (m)	220 [0, 350]	222 [0, 356]	216 [0, 350]	160 [0, 328]	0.685
LVEF (%)	30 [25, 36]	30 [25, 35]	30 [25, 37]	33 [25, 40]	0.052
LVEF > 40%	216 (10.7%)	122 (9.8%)	77 (11.7%)	17 (16.7%)	0.062
Laboratory indices					
NT-proBNP (pg/mL)	2721.0 [1204.3, 5741.8]	2608.5 [1204.3, 5337.5]	2798.0 [1170.0, 6460.0]	3144.0 [1513.0, 6073.0]	0.289
CRP (mg/L)	13.0 [5.8, 26.4]	12.5 [5.8, 26.4]	13.5 [5.8, 26.6]	15.1 [4.8, 26.6]	0.711
eGFR (mL/min/1.73 m^2^)	65.0 (26.2)	65.5 (25.7)	64.8 (26.6)	60.1 (29.2)	0.108
Creatinine (µmol/L)	101.0 [82.1, 128.2]	100.0 [81.0, 125.0]	102.0 [84.0, 132.6]	106.1 [88.1, 151.5]	**0**.027*
Hb (g/dL)	13.2 (1.9)	13.2 (1.9)	13.2 (1.9)	12.8 (1.9)†	**0**.047*
Medications					
BB	1880 (83.4%)	1159 (83.0%)	624 (84.0%)	97 (85.1%)	0.737
BB at target dose	127 (5.6%)	74 (5.3%)	43 (5.8%)	10 (8.8%)	0.295
BB fraction of target dose	0.25 [0.06, 0.50]	0.25 [0.06, 0.48]	0.25 [0.06, 0.50]	0.25 [0.06, 0.50]	0.527
ACEi/ARB	1615 (71.7%)	1011 (72.4%)	531 (71.5%)	73 (64.0%)	0.163
ACEi/ARB at target dose	295 (13.1%)	181 (13.0%)	99 (13.3%)	15 (13.2%)	0.971
ACEi/ARB fraction of target dose	0.25 [0.00, 0.50]	0.25 [0.00, 0.50]	0.25 [0.00, 0.50]	0.25 [0.00, 0.50]	0.267
MRA	1196 (53.1%)	766 (54.8%)	379 (51.0%)	51 (44.7%)	**0**.045*
Digoxin	424 (18.8%)	259 (18.5%)	149 (20.1%)	16 (14.0%)	0.283
Oral hypoglycaemic agents**	457 (62.4%)	290 (63.2%)	150 (63.3%)	17 (47.2%)	0.155

b.p.m., beats per minute; HF, heart failure; AF, atrial fibrillation; DM, diabetes mellitus; HT, hypertension; RD, renal disease; COPD, chronic obstructive pulmonary disease; MI, myocardial infarction; NYHA, New York Heart Association; BMI, body mass index; HR, heart rate; SBP/DBP, systolic/diastolic blood pressure; 6MWT, 6-min walk test; LVEF, left ventricular ejection fraction; NT-proBNP, N-terminal pro-brain natriuretic peptide; CRP, C-reactive protein; Hb, haemoglobin; BB, β-adrenoreceptor antagonist; ACEi, angiotensin-converting enzyme inhibitor; ARB, angiotensin receptor blocker; MRA, mineralocorticoid receptor antagonist. *P ≤ 0.05; **only for patients with diabetes mellitus; †significantly different from seronegative patients in post hoc testing.

**Table 2:** Baseline characteristics stratified according to anti-β2-adrenergic receptor autoantibody status

Variable	Anti-β2 seronegative	Anti-β2 intermediate	Anti-β2 seropositive	*P*-value
Group size	1326	797	133	N/A
Demographics				
Age (years)	69 (12)†	68 (13)	69 (12)	**0**.046*
Female sex	373 (28.2%)	190 (23.9%)	39 (29.3%)	0.076
Recruited as outpatient	458 (34.6%)	223 (28.0%)	53 (39.8%)	**0**.001*
Primary HF aetiology				
Cardiomyopathy	325 (24.9%)	197 (25.3%)	34 (26.0%)	0.96
Hypertensive	132 (10.1%)	80 (10.3%)	21 (16.0%)	0.106
Ischaemic	606 (46.5%)	359 (46.1%)	53 (40.5%)	0.415
Valvular disease	96 (7.4%)	62 (8.0%)	9 (6.9%)	0.846
Previous history and comorbidities				
AF	608 (45.9%)	355 (44.6%)	61 (45.9%)	0.842
DM	451 (34.0%)	242 (30.4%)	39 (29.3%)	0.162
HT	828 (62.5%)	485 (60.9%)	85 (63.9%)	0.695
RD	375 (28.3%)	218 (27.4%)	35 (26.3%)	0.829
Anaemia	438 (36.4%)	269 (36.8%)	39 (32.8%)	0.691
COPD	235 (17.7%)	130 (16.3%)	30 (22.6%)	0.207
MI	525 (39.6%)	299 (37.6%)	40 (30.1%)	0.083
Stroke	129 (9.7%)	63 (7.9%)	20 (15.0%)	**0**.027*
HF hospitalization during previous year	419 (31.6%)	252 (31.7%)	40 (30.1%)	0.932
Smoking				**0**.021*
Never	494 (37.4%)	284 (35.7%)	46 (34.6%)	
Past	665 (50.3%)	380 (47.7%)	60 (45.1%)	
Current	163 (12.3%)	132 (16.6%)	27 (20.3%)	
Clinical characteristics				
NYHA functional class				
I	121 (10.5%)	79 (11.4%)	9 (7.8%)	0.32
II	602 (52.2%)	348 (50.1%)	64 (55.7%)	
III	377 (32.7%)	246 (35.4%)	40 (34.8%)	
IV	54 (4.7%)	22 (3.2%)	2 (1.7%)	
NYHA class III or IV	431 (37.3%)	268 (38.6%)	42 (36.5%)	0.841
BMI (kg/m^2^)	27.2 [24.2, 30.9]	27.1 [24.2, 30.4]	26.6 [23.5, 29.5]	0.104
HR (b.p.m.)	76 [66, 89]	77 [68, 90]	75 [65, 87]	0.158
SBP (mm Hg)	125 (22)	124 (22)	127 (23)	0.221
DBP (mm Hg)	75 (13)	75 (13)	75 (14)	0.95
Pulmonary crackles	669 (52.2%)	417 (53.6%)	70 (54.3%)	0.787
6MWT successfully completed	837 (65.5%)	501 (64.9%)	75 (57.7%)	0.203
6MWT distance (m)	220 [0, 350]	224 [0, 356]	161 [0, 326]	0.237
LVEF (%)	30 [25, 37]	30 [24, 35]	30 [25, 35]	**0**.036*
LVEF > 40%	145 (12.2%)	60 (8.5%)	11 (9.2%)	**0**.034*
Laboratory indices				
NT-proBNP (pg/mL)	2798.0 [1226.5, 5738.0]	2678.5 [1200.5, 5803.5]	2542.0 [905.5, 5606.0]	0.885
CRP (mg/L)	13.4 [5.8, 26.7]	12.5 [5.9, 24.9]	11.9 [3.7, 26.6]	0.379
eGFR (mL/min/1.73 m^2^)	64.2 (25.4)	66.0 (27.3)	66.2 (28.0)	0.275
Creatinine (µmol/L)	101.7 [82.0, 128.0]	101.3 [83.1, 131.9]	96.8 [80.0, 118.8]	0.638
Hb (g/dL)	13.2 (1.9)	13.2 (1.9)	13.4 (1.8)	0.512
Medications				
BB	1107 (83.5%)	660 (82.9%)	113 (85.0%)	0.823
BB at target dose	79 (6.0%)	39 (4.9%)	9 (6.8%)	0.497
BB fraction of target dose	0.25 [0.06, 0.50]	0.25 [0.06, 0.38]	0.25 [0.08, 0.38]	0.617
ACEi/ARB	941 (71.0%)	569 (71.5%)	105 (78.9%)	0.153
ACEi/ARB at target dose	170 (12.8%)	106 (13.3%)	19 (14.3%)	0.869
ACEi/ARB fraction of target dose	0.25 [0.00, 0.50]	0.25 [0.00, 0.50]	0.25 [0.12, 0.50]	0.099
MRA	677 (51.1%)	458 (57.5%)	61 (45.9%)	**0**.004*
Digoxin	252 (19.0%)	149 (18.7%)	23 (17.3%)	0.886
Oral hypoglycaemic agents**	272 (60.3%)	165 (68.2%)	20 (51.3%)	**0**.042*

HF, heart failure; AF, atrial fibrillation; DM, diabetes mellitus; HT, hypertension; RD, renal disease; COPD, chronic obstructive pulmonary disease; MI, myocardial infarction; NYHA, New York Heart Association; BMI, body mass index; HR, heart rate; SBP/DBP, systolic/diastolic blood pressure; 6MWT, 6-min walk test; LVEF, left ventricular ejection fraction; NT-proBNP, N-terminal pro-brain natriuretic peptide; CRP, C-reactive protein; Hb, haemoglobin; BB, β-adrenoreceptor antagonist; ACEi, angiotensin-converting enzyme inhibitor; ARB, angiotensin receptor blocker; MRA, mineralocorticoid receptor antagonist. *P ≤ 0.05; **only for patients with diabetes mellitus; †significantly different from intermediate patients in post hoc testing.

**Table 3:** Baseline characteristics stratified according to anti-β3-adrenergic receptor autoantibody status

Variable	Anti-β3 seronegative	Anti-β3 intermediate	Anti-β3 seropositive	*P*-value
Group size	1575	640	41	N/A
Demographics				
Age (years)	69 (12)	69 (12)	71 (11)	0.346
Female sex	430 (27.3%)	160 (25.0%)	12 (29.3%)	0.495
Recruited as outpatient	504 (32.0%)	213 (33.3%)	17 (41.5%)	0.402
Primary HF aetiology				
Cardiomyopathy	393 (25.5%)	156 (24.6%)	7 (17.9%)	0.532
Hypertensive	154 (10.0%)	76 (12.0%)	3 (7.7%)	0.322
Ischaemic	716 (46.5%)	284 (44.9%)	18 (46.2%)	0.794
Valvular disease	113 (7.3%)	50 (7.9%)	4 (10.3%)	0.732
Previous history and comorbidities				
AF	687 (43.7%)	316 (49.4%)	21 (51.2%)	**0**.038*
DM	516 (32.8%)	202 (31.6%)	14 (34.1%)	0.83
HT	988 (62.8%)	385 (60.2%)	25 (61.0%)	0.502
RD	419 (26.6%)	196 (30.6%)	13 (31.7%)	0.142
Anaemia	513 (35.5%)	222 (38.9%)	11 (29.7%)	0.248
COPD	260 (16.5%)	123 (19.2%)	12 (29.3%)	**0**.044*
MI	611 (38.8%)	241 (37.7%)	12 (29.3%)	0.423
Stroke	151 (9.6%)	55 (8.6%)	6 (14.6%)	0.391
HF hospitalization during previous year	482 (30.6%)	215 (33.6%)	14 (34.1%)	0.374
Smoking				0.453
Never	575 (36.6%)	236 (37.0%)	13 (31.7%)	
Past	765 (48.7%)	321 (50.3%)	19 (46.3%)	
Current	232 (14.8%)	81 (12.7%)	9 (22.0%)	
Clinical characteristics				
NYHA functional class				
I	142 (10.4%)	64 (11.3%)	3 (8.3%)	0.599
II	717 (52.6%)	276 (48.9%)	21 (58.3%)	
III	448 (32.8%)	205 (36.3%)	10 (27.8%)	
IV	57 (4.2%)	19 (3.4%)	2 (5.6%)	
NYHA class III or IV	505 (37.0%)	224 (39.7%)	12 (33.3%)	0.465
BMI (kg/m^2^)	27.2 [24.2, 30.8]	26.9 [23.8, 30.2]	26.6 [24.2, 29.1]	0.224
HR (b.p.m.)	76 [67, 89]	76 [67, 90]	72 [63, 87]	0.494
SBP (mm Hg)	125 (22)	124 (22)	123 (19)	0.609
DBP (mm Hg)	75 (13)	75 (13)	73 (11)	0.432
Pulmonary crackles	789 (51.5%)	344 (55.8%)	23 (56.1%)	0.178
6MWT successfully completed	997 (65.4%)	394 (64.1%)	22 (56.4%)	0.456
6MWT distance (m)	225 [0, 360]	215 [0, 344]	197 [0, 314]	0.39
LVEF (%)	30 [25, 36]	30 [24, 36]	30 [25, 35]	0.553
LVEF > 40%	147 (10.5%)	64 (11.2%)	5 (13.9%)	0.737
Laboratory indices				
NT-proBNP (pg/mL)	2600.0 [1158.0, 5581.0]	2950.0 [1296.5, 5995.5]	2685.5 [1356.5, 5958.8]	0.153
CRP (mg/L)	12.8 [5.7, 26.2]	13.2 [6.0, 26.8]	19.2 [6.9, 32.6]	0.23
eGFR (mL/min/1.73 m^2^)†	65.9 (26.6)	63.0 (24.9)	60.5 (31.3)	**0**.032*
Creatinine (µmol/L)	100.0 [80.9, 126.0]	102.8 [86.0, 133.0]	112.0 [84.0, 140.6]	**0**.027*
Hb (g/dL)	13.2 (1.9)	13.1 (2.0)	13.4 (1.8)	0.401
Medications				
BB	1307 (83.1%)	538 (84.1%)	35 (85.4%)	0.808
BB at target dose	84 (5.3%)	36 (5.6%)	7 (17.1%)	**0**.006*
BB fraction of target dose	0.25 [0.06, 0.48]	0.25 [0.08, 0.50]	0.25 [0.05, 0.50]	0.351
ACEi/ARB	1137 (72.3%)	446 (69.7%)	32 (78.0%)	0.309
ACEi/ARB at target dose	201 (12.8%)	87 (13.6%)	7 (17.1%)	0.654
ACEi/ARB fraction of target dose	0.25 [0.00, 0.50]	0.25 [0.00, 0.50]	0.25 [0.12, 0.50]	0.324
MRA	835 (53.1%)	339 (53.0%)	22 (53.7%)	0.996
Digoxin	293 (18.6%)	122 (19.1%)	9 (22.0%)	0.85
Oral hypoglycaemic agents**	322 (62.4%)	127 (62.9%)	8 (57.1%)	0.912

HF, heart failure; AF, atrial fibrillation; DM, diabetes mellitus; HT, hypertension; RD, renal disease; COPD, chronic obstructive pulmonary disease; MI, myocardial infarction; NYHA, New York Heart Association; BMI, body mass index; HR, heart rate; SBP/DBP, systolic/diastolic blood pressure; 6MWT, 6-min walk test; LVEF, left ventricular ejection fraction; NT-proBNP, *N*-terminal pro-brain natriuretic peptide; CRP, C-reactive protein; Hb, haemoglobin; BB, β-adrenoreceptor antagonist; ACEi, angiotensin-converting enzyme inhibitor; ARB, angiotensin receptor blocker; MRA, mineralocorticoid receptor antagonist. *P ≤ 0.05; **only for patients with diabetes mellitus; †no significant between-group differences in post hoc testing.

**Table 4:** Baseline characteristics stratified according to anti-M2-muscarinic receptor autoantibody status

Variable	Anti-M2 seronegative	Anti-M2 intermediate	Anti-M2 seropositive	*P*-value
Group size	1213	871	172	N/A
Demographics				
Age (years)	69 (12)	69 (13)	70 (11)	0.260
Female sex	338 (27.9%)	215 (24.7%)	49 (28.5%)	0.224
Recruited as outpatient	403 (33.1%)	276 (32.0%)	55 (32.0%)	0.862
Primary HF aetiology				
Cardiomyopathy	301 (25.3%)	214 (25.1%)	41 (24.0%)	0.935
Hypertensive	116 (9.7%)	102 (12.0%)	15 (8.8%)	0.195
Ischaemic	539 (45.3%)	399 (46.9%)	80 (46.8%)	0.750
Valvular disease	87 (7.3%)	66 (7.8%)	14 (8.2%)	0.881
Previous history and comorbidities				
AF	540 (44.6%)	400 (45.9%)	84 (48.8%)	0.539
DM	376 (31.0%)	296 (34.0%)	60 (34.9%)	0.289
HT	733 (60.5%)	555 (63.7%)	110 (64.0%)	0.289
RD	329 (27.2%)	255 (29.3%)	44 (25.6%)	0.449
Anaemia	409 (36.9%)	286 (36.1%)	51 (33.6%)	0.704
COPD	204 (16.8%)	152 (17.5%)	39 (22.7%)	0.170
MI	454 (37.5%)	345 (39.6%)	65 (37.8%)	0.611
Stroke	122 (10.1%)	79 (9.1%)	11 (6.4%)	0.275
HF hospitalization during previous year	383 (31.4%)	276 (32.0%)	52 (30.2%)	0.895
Smoking				0.308
Never	436 (36.0%)	332 (38.2%)	56 (32.6%)	
Past	606 (50.1%)	405 (46.6%)	94 (54.7%)	
Current	168 (13.9%)	132 (15.2%)	22 (12.8%)	
Clinical characteristics				
NYHA functional class				
I	109 (10.4%)	84 (11.0%)	16 (10.5%)	0.518
II	561 (53.4%)	370 (48.6%)	83 (54.2%)	
III	339 (32.3%)	277 (36.4%)	47 (30.7%)	
IV	41 (3.9%)	30 (3.9%)	7 (4.6%)	
NYHA class III or IV	380 (36.2%)	307 (40.3%)	54 (35.3%)	0.161
BMI (kg/m^2^)	27.0 [24.0, 30.5]	27.2 [24.2, 30.8]	27.3 [24.5, 30.5]	0.382
HR (b.p.m.)	76 [66, 90]	76 [67, 90]	75 [69, 88]	0.898
SBP (mm Hg)	125 (23)	124 (21)	123 (20)	0.131
DBP (mm Hg)	75 (14)	75 (13)	74 (12.2)	0.407
Pulmonary crackles	615 (52.3%)	454 (54.0%)	87 (50.6%)	0.634
6MWT successfully completed	765 (65.3%)	552 (65.6%)	96 (57.8%)	0.142
6MWT distance (m)	230 [0, 350]	217 [0, 350]	167 [0, 360]	0.539
LVEF (%)	30 [25, 37]	30 [25, 36]	30 [25, 35]	0.832
LVEF > 40%	119 (11.1%)	82 (10.6%)	15 (9.3%)	0.775
Laboratory indices				
NT-proBNP (pg/mL)	2711.0 [1157.5, 5460.0]	2879.0 [1268.0, 6066.0]	2356.0 [1231.8, 5396.8]	0.253
CRP (mg/L)	13.1 [5.7, 25.0]	13.0 [5.9, 27.1]	13.2 [5.5, 27.1]	0.847
eGFR (mL/min/1.73 m^2^)	65.4 (25.8)	64.4 (26.8)	65.0 (26.3)	0.664
Creatinine (µmol/L)	100.0 [82.0, 126.4]	104.2 [83.0, 132.6]	99.5 [83.7, 132.2]	0.167
Hb (g/dL)	13.2 (1.9)	13.2 (1.9)	13.3 (2.0)	0.826
Medications				
BB	1020 (84.2%)	715 (82.1%)	145 (84.3%)	0.410
BB at target dose	69 (5.7%)	45 (5.2%)	13 (7.6%)	0.457
BB fraction of target Dose	0.25 [0.06, 0.50]	0.24 [0.06, 0.48]	0.25 [0.06, 0.50]	0.347
ACEi/ARB	866 (71.5%)	625 (71.8%)	124 (72.1%)	0.984
ACEi/ARB at target dose	157 (13.0%)	105 (12.1%)	33 (19.2%)	**0**.040*
ACEi/ARB fraction of target dose	0.25 [0.00, 0.50]	0.25 [0.00, 0.50]	0.25 [0.00, 0.50]	0.956
MRA	615 (50.8%)	477 (54.8%)	104 (60.5%)	**0**.026*
Digoxin	223 (18.4%)	169 (19.4%)	32 (18.6%)	0.848
Oral hypoglycaemic agents**	227 (60.4%)	191 (64.5%)	39 (65.0%)	0.496

HF, heart failure; AF, atrial fibrillation; DM, diabetes mellitus; HT, hypertension; RD, renal disease; COPD, chronic obstructive pulmonary disease; MI, myocardial infarction; NYHA, New York Heart Association; BMI, body mass index; HR, heart rate; SBP/DBP, systolic/diastolic blood pressure; 6MWT, 6-min walk test; LVEF, left ventricular ejection fraction; NT-proBNP, *N*-terminal pro-brain natriuretic peptide; CRP, C-reactive protein; Hb, haemoglobin; BB, β-adrenoreceptor antagonist; ACEi, angiotensin-converting enzyme inhibitor; ARB, angiotensin receptor blocker; MRA, mineralocorticoid receptor antagonist. *P ≤ 0.05; **only for patients with diabetes mellitus.

#### 3.3.1 Anti-β1 autoantibodies

Anti-β1 AAB seropositivity was associated with a lower prevalence of a primary cardiomyopathy aetiology [seronegative vs. intermediate vs. seropositive: 372 (27.1%) vs. 169 (23.2%) vs. 15 (13.4%), *P* = 0.002], a higher prevalence of a history of renal disease [366 (26.2%) vs. 216 (29.1%) vs. 46 (40.1%), *P* = 0.004], anaemia [446 (35.1%) vs. 250 (37.0%) vs. 50 (47.2%), *P* = 0.043], and chronic obstructive pulmonary disease (COPD) [232 (16.6%) vs. 133 (17.9%) vs. 30 (26.3%), *P* = 0.030]. These were also mirrored by corresponding changes in serum creatinine and haemoglobin (*P* = 0.027 and 0.047, respectively). Lastly, seropositivity was associated with a lower prevalence of mineralocorticoid receptor antagonist (MRA) use [766 (54.8%) vs. 379 (51.0%) vs. 51 (44.7%), *P* = 0.045]. Amongst patients with a primary ischaemic HF aetiology, β1-seropositivity did not differ significantly amongst those with and without a history of myocardial infarction (MI) [40 (5.2%) vs. 17 (7.0%), *P* = 0.514].

#### 3.3.2 Anti-β2 autoantibodies

Anti-β2 AAB seropositivity was associated with a history of stroke [129 (9.7%) vs. 63 (7.9%) vs. 20 (15.0%), *P* = 0.027] and current smoking (*P* = 0.021). The proportion of seropositive patients with an LVEF ≥ 40% was modestly lower compared to seronegative patients [145 (12.2%) vs. 60 (8.5%) vs. 11 (9.2%), *P* = 0.034]. Seropositive patients were more likely to have been recruited as outpatients rather than inpatients [458 (34.6%) vs. 223 (28.0%) vs. 53 (39.8%), *P* = 0.001]. Similar to anti-β1 seropositive patients, anti-β2 seropositivity was associated with a lower prevalence of MRA use [677 (51.1) vs. 458 (57.5%) vs. 61 (45.9%), *P* = 0.004]. Seropositive diabetics were also less likely to be using oral hypoglycaemic agents (*P* = 0.042).

#### 3.3.3 Anti-β3 autoantibodies

Anti-β3 AAB seropositivity was associated with a higher prevalence of atrial fibrillation (AF) and COPD [687 (43.7%) vs. 316 (49.4%) vs. 21 (51.2%), *P* = 0.038, and 260 (16.5%) vs. 123 (19.2%) vs. 12 (29.3%), *P* = 0.044, respectively]. In addition, mean eGFR was lower in seropositive patients [65.92 (26.57) vs. 62.98 (24.91) vs. 60.50 (31.33), *P* = 0.032], with corresponding increases in serum creatinine (*P* = 0.027). Lastly, a greater proportion of seropositive patients was at the guideline-recommended target dose for BB [84 (5.3%) vs. 36 (5.6%) vs. 7 (17.1%), *P* = 0.006].

#### 3.3.4 Anti-M2 autoantibodies

Seropositivity for anti-M2 AABs was associated with a higher probability of being at the guideline-recommended target dose for ACEi [157 (13.0%) vs. 105 (12.1%) vs. 33 (19.2%), *P* = 0.040], as well as higher probability of MRA use [615 (50.8%) vs. 477 (54.8%) vs. 104 (60.5%), *P* = 0.026].

### 3.4 Survival analysis

**Figure 3:**
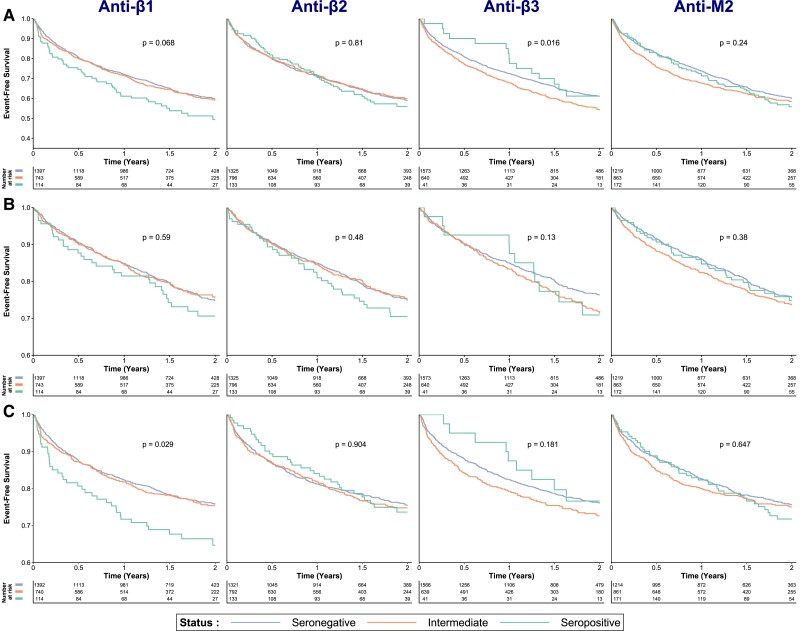
Kaplan–Meier curves for the combined outcome (all-cause mortality and rehospitalization for HF) censored at 2 years (*A*) and for all-cause mortality alone (*B*), as well as cumulative incidence function curves for rehospitalization for HF only with all-cause mortality as a competing risk (*C*). From left to right, results of univariable analyses for anti-β1, anti-β2, anti-β3, and anti-M2 autoantibodies. HF, heart failure.

**Figure 4:**
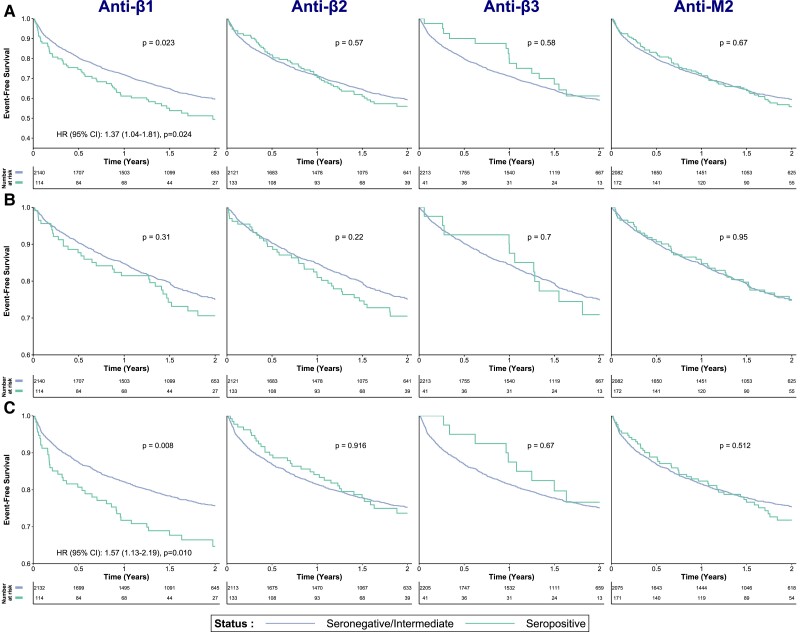
Sensitivity analyses of the results presented in *Figure 3*, by stratifying categories as seronegative/intermediate and seropositive. Kaplan–Meier curves for the combined outcome (all-cause mortality and rehospitalization for HF) censored at 2 years (*A*) and for all-cause mortality alone (*B*), as well as cumulative incidence function curves for rehospitalization for HF only with all-cause mortality as a competing risk (*C*). For anti-β1 autoantibodies, Cox regression analyses for the combined outcome and competing risks analyses for HF-related rehospitalization (both univariable and multivariable) are presented in the *Results* section. HF, heart failure.

Kaplan–Meier survival curves are presented for each AAB in *Figure 3A* for the combined outcome and all-cause mortality alone (*Figure 3B*) at 2-year follow-up. Cumulative incidence function curves for the competing risk analysis of HF rehospitalization at 2-year follow-up with all-cause mortality as a competing risk are presented in *Figure 3C*. Regarding the primary outcome, anti-β1 seropositive patients trended towards a poorer prognosis, without reaching statistical significance (*P* = 0.068). Anti-β3 status was associated with the primary outcome, with seropositive patients having an overall better prognosis (*P* = 0.016). Anti-β2 and anti-M2 status were not significantly associated with the primary outcome. There were no significant associations between AAB status and all-cause mortality. Competing risks analysis revealed only a significant association between anti-β1 seropositivity and a higher probability of HF rehospitalization (*P* = 0.029). A sensitivity analysis was performed after merging seronegative and intermediate patients because these groups showed comparable results in previous survival analyses. This revealed that the primary outcome and HF rehospitalization alone were more frequent only in patients who were seropositive for anti-β1 AABs (*P* = 0.023 and 0.008, respectively) (*Figure 4*).

Since the two significant comparisons for anti-β1 AABs in the sensitivity analysis also met the criteria for the proportionality of hazards assumption, additional multivariable analyses were performed (Cox regression for the combined outcome and competing risk regression for rehospitalization). Compared with seronegative/intermediate status, anti-β1 seropositivity was associated with the combined outcome [hazard ratio (HR), 95% confidence interval (95% CI): 1.37 (1.04–1.81), *P* = 0.024] but ceased to be significant when corrected for the previously published BIOSTAT-CHF risk model [HR (95% CI): 1.27 (0.97–1.68), *P* = 0.087]. Anti-β1 seropositivity was associated with HF rehospitalization both in univariable analysis [HR (95%CI): 1.57 (1.13–2.19), *P* = 0.010] and when adjusted for the BIOSTAT-CHF risk model [HR (95% CI): 1.47 (1.05–2.07), *P* = 0.030]. Additional validation was performed by means of propensity score matching as described in the *Methods* section. All patients that were anti-β1 seropositive except one (*n* = 113) were matched 1 : 1 to seronegative patients, forming well-balanced groups (standardized mean differences < 0.1, variance ratios < 2). Seropositive patients had significantly higher risk for HF rehospitalization at 2-year follow-up (standardized HR (95% CI) 1.83 (1.08–3.10), *P* = 0.024).

Because patients with intermediate AAB status seemed to have a different prognosis, particularly in the case of anti-β3 AABs (*Figure 3*), additional analyses were performed in patients with intermediate AAB status for all AABs, in order to investigate whether the AAB titre as a continuous variable was associated with any of the examined outcomes (see Supplementary material online, *Table S3*). Intermediate patients for anti-β3 AABs showed an inverse association between anti-β3 AAB titre and the combined outcome [HR (95% CI): 0.82 (0.71–0.94) for each one-unit increase, *P* = 0.006], which remained significant after adjustment for the corresponding risk model [HR (95% CI): 0.83 (0.72–0.95), *P* = 0.009]. In patients with intermediate anti-β2 status, AAB titres were associated with reduced all-cause mortality [HR (95% CI): 0.83 (0.72–0.95), *P* = 0.008]. However, this was no longer significant after multivariable correction for the corresponding risk model (*P* = 0.196). Patients with intermediate status for anti-β1/anti-M2 AABs did not show associations between their corresponding AAB titres and any examined outcomes.

### 3.5 Analysis of circulating B-lymphocyte-associated markers in relation to autoantibody seropositivity

**Figure 5:**
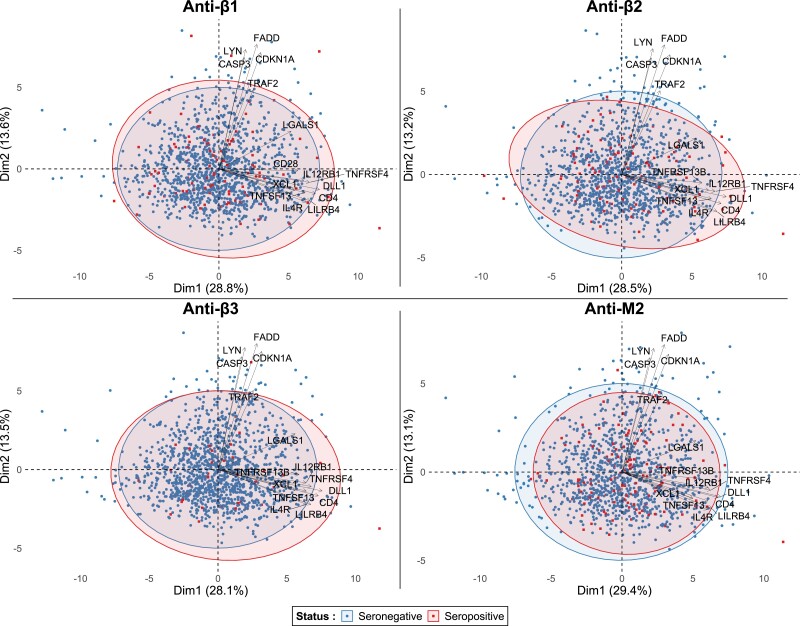
Biplot of principal component analysis of the 31 biomarkers involved in B-lymphocyte-associated biological processes. The intermediate group for each AAB was excluded to accentuate potential differences. Considerable overlap is seen between groups in all comparisons. For optimal visualization, only the top 15 biomarkers that contributed the most to the first principal component are shown. The relative contribution of all examined biomarkers is shown in Supplementary material online, *Figure S3*). AAB, autoantibody.

PCA was used on the 31 B-lymphocyte-related biomarkers (*Figure 5*). Concentration ellipses were plotted for seropositive and seronegative individuals per AAB. All sub-analyses demonstrated considerable overlap between seropositive and seronegative patients. The relative contribution of the 31 biomarkers to the first and second principal components is presented in Supplementary material online, *Figure S3*.

## 4. Discussion

In this post hoc analysis of a large and heterogeneous cohort of patients with HF, 16.9% of patients were seropositive for at least one ANS-AAB. Overall, the prevalence of seropositivity did not significantly differ from that of healthy controls, with the exception of a significantly higher prevalence of seropositivity for anti-M2 AABs. Seropositivity for ANS-AABs was associated with the presence of comorbidities (renal disease, COPD, AF, and stroke) or ARB/MRA use. Only seropositivity for anti-β1 AABs was significantly associated with a higher probability of HF rehospitalization at 2-year follow-up. PCA revealed considerable overlap of B-lymphocyte activity between seropositive and seronegative patients with HF, based on 31 circulating biomarkers.

Most studies evaluating ANS-AABs in patients with HF have been conducted in patients with Chagas cardiomyopathy and non-hereditary DCM, mostly focusing on anti-β1 and anti-M2 AABs. Approximately a third of patients with asymptomatic Chagas disease have detectable anti-β1, anti-β2, and anti-M2 AABs, while almost all who develop cardiomyopathy are seropositive for all aforementioned AABs.^8^ Amongst patients with non-hereditary DCM, anti-β1 and anti-M2 AABs were detected in 31% and 36–39% of patients, respectively.^14^ Interestingly, immunoglobulin subclasses may also be relevant, as in patients with DCM, the presence of IgG3-anti-β1 AABs was associated with a better LVEF during follow-up compared with seronegative patients, or those with non-IgG3-anti-β1 AABs.^15^ Furthermore, an increased prevalence of ANS-AAB seropositivity has been reported in women with peripartum cardiomyopathy (PPCM) compared with healthy pregnant controls.^16^ In patients with non-hereditary DCM, anti-β1 AABs are also associated with an increased risk of ventricular tachycardia and sudden cardiac death,^17^ while anti-M2 AABs predict rehospitalization but not all-cause mortality in PPCM.^18^ In our study, we only identified an independent association between seropositivity for anti-β1 AABs with HF rehospitalization at 2-year follow-up, similar to a previous study in a smaller cohort.^19^ This might be explained by counterbalance of anti-β1 AAB actions by anti-β2 AABs,^20^ although their co-occurrence was limited. Conversely, seropositivity for anti-β3 AABs trended towards an association with better prognosis. However, the low seroprevalence of anti-β3 AABs made additional analyses difficult.

ANS-AABs can potentially influence cardiac function by causing inappropriate positive or negative chronotropic or inotropic effects, by inducing cardiomyocyte apoptosis, or by activating the complement cascade.^14,21–23^ Other authors reported that anti-β1 AABs from patients with DCM enhanced T-cell proliferation via PKA/MAPK-signalling, which was prevented by metoprolol.^24^ They also reduced interferon-γ production, while increasing IL-4 production by T-cells.^24^ Anti-β1 AABs also impaired endothelial function in Wistar rats by negatively affecting NO signaling.^25^ In contrast, anti-β3 AABs from patients with HF protected rats with abdominal aortic banding from developing LV dysfunction and dilation.^26^ Anti-β1 and anti-M2 AABs have been found to induce HF in rodents, which could be reversed upon their removal.^27,28^ This suggests a drug-like function on their corresponding target receptors.^28^ However, the pharmacology governing these interactions is as of yet unclear^28^ and the agonist or antagonist characteristics of anti-β1 AABs can also be of interest with regard to whether their removal would lead to clinical benefit.^27^

Extensive studies in the setting of autoimmunity have led to the identification of numerous mechanisms underlying the generation of AABs. One such mechanism has been termed ‘molecular mimicry’, which describes the process of antibody generation against microbial proteins, which are also structurally homologous to self-peptides.^29^ A search of the literature did not reveal the presence of any structural homologues of ANS receptors; still, structural similarities between microbial peptides and ANS receptors cannot be ruled out. A different mechanism involves the generation of AABs against strictly intracellular antigens, as often occurs in autoimmune diseases (e.g. anti-nuclear antibodies and anti-cytoplasmic antibodies).^30^ The latter is less likely in this case, considering that ANS receptors are transported to the cell membrane. An additional explanation could be that B-lymphocytes expressing B-cell receptors that recognize self-antigens with high affinity are efficiently depleted or functionally silenced, while B-lymphocytes with medium- or low-affinity receptors may escape tolerance mechanisms and give rise to antibody-producing plasma cells.^31^ Collectively, circulating markers of B-lymphocyte function did not differ significantly between seropositive and seronegative patients in our PCA analyses, suggesting that ANS-AAB generation was not an acute process.

Autoreactive B-lymphocytes and plasma cells also occur in the general population, and seropositivity for AABs associated with thyroid disorders or autoimmune diseases does not per se lead to clinical manifestations.^14,32,33^ Yet, both within the field of cardiovascular disease and in other disease states, circulating ANS-AABs have been implicated in the pathophysiology of various diseases. The reasons behind the differential effects of circulating AABs in the general population and in specific disease processes remain unknown. Natural autoreactive immunoglobulin-M (IgM) antibodies may protect from autoimmune disease,^34^ while the subtype of circulating IgG antibodies as well as the glycosylation and sialylation status of their Fc-segments regulates higher affinity binding to inhibitory Fcγ receptors (FcγRIIA, FcγRIIB).^31^ Interestingly, animal studies have shown that the generation of IgG antibodies with pro-inflammatory profiles requires a T-lymphocyte-dependent immune response,^35^ which is in agreement with previous findings by our group underlying the important prognostic role of T-lymphocyte activation in HF.^10^ Furthermore, anti-β1 AABs from patients with HF lead to cardiac fibrosis, cardiomyocyte apoptosis, LV dilatation, and increases in LV mass in wild-type mice, which was not observed in mice lacking T-lymphocytes.^36^ The same study showed that blockade of IL-6 production using small interfering RNA in wild-type mice ameliorated cardiomyocyte apoptosis and that anti-β1 AABs upregulated IL-6 production in T-lymphocytes from patients with HF.^36^ These findings illustrate the interplay between T-lymphocytes and ANS-AABs and are further supported by the prognostic value of IL-6 in HF.^37^

### 4.1 Limitations

The post hoc character of this study and the relatively low prevalence of AAB seropositivity suggest that some analyses may have been underpowered, particularly in the case of anti-β3 AABs. Additional characteristics for the healthy control population were not available and could thus not be accounted for in statistical analyses. Additionally, the control population was significantly different from the examined patients (younger, more women), and our findings could suggest potential age dependency of AAB formation. Extensive antibody repertoire mapping was not performed, which might have led to the identification of AABs with greater prognostic implications. In addition, our study did not include any patients with Chagas cardiomyopathy or PPCM and lacked the necessary data to determine the presence of non-hereditary DCM. The BIOSTAT-CHF cohort included patients that were suboptimally treated for HF and might thus not be representative of the general HF population. Furthermore, it could be hypothesized that the uptitration of HF treatments after initial blood sampling could have masked or attenuated the effects of AABs on outcomes. Lastly, we did not evaluate the functional characteristics of measured AABs (e.g. inhibitory and stimulatory), and the specific isotype/class, subclass, and allotype of AABs were not determined.

### 4.2 Conclusion

In a large and heterogeneous population of patients with HF, seropositivity for ANS-AABs was not strongly associated with adverse outcomes and was mostly related to existing comorbidities and medication use, while only seropositivity for anti-β1 AABs was independently associated with rehospitalization for HF. Further studies are necessary to better elucidate the potential prognostic significance and clinical associations of ANS-AABs in patients with non-hereditary DCM, Chagas cardiomyopathy, or PPCM.

## Supplementary material

Supplementary material is available at *Cardiovascular Research* online.

## Authors’ contribution

G.M.-M. (design, analysis, interpretation, drafting, and revision), W.B.M. (analysis and revision), A.A.A.-M. (design, analysis, interpretation, and revision), S.D.A. (acquisition and revision), J.G.F.C. (acquisition and revision), K.D. (acquisition and revision), C.C.L. (acquisition and revision), L.L.N. (acquisition and revision), N.J.S. (acquisition and revision), F.Z. (acquisition and revision), M.M. (acquisition and revision), P.S. (design, acquisition, analysis, and revision), A.H. (design, acquisition, analysis, and revision), P.L. (design, acquisition, analysis, and revision), D.J.v.V. (acquisition and revision), R.A.d.B. (acquisition and revision), A.A.V. (acquisition and revision), P.v.d.M. (acquisition and revision), L.S. (conception, design, analysis, interpretation, and revision), and N.B. (conception, acquisition, analysis, interpretation, and revision).

## Supplementary Material

cvad042_Supplementary_Data

## Data Availability

Data are available upon reasonable request to the corresponding author.

## Notes

Translational perspective Currently, little is known regarding the clinical/prognostic associations of autoantibodies against autonomic nervous system receptors (ANS-AABs) in patients with heart failure (HF). We show that out of four investigated ANS-AABs, only anti-β1 AAB seropositivity was independently associated with HF rehospitalization. Seropositivity for any of the investigated AAB was not associated with changes in circulating B-lymphocyte-related markers. Seropositivity was highly similar when compared to healthy controls. These findings support a limited prognostic role for ANS-AABs in the general HF population and suggest that ANS-AAB generation may constitute a previous event unrelated to HF. Future studies should investigate specific target groups such as Chagas cardiomyopathy, non-hereditary dilated cardiomyopathy (DCM), and peripartum cardiomyopathy (PPCM).
