# Supplementary material for: Clinical and prognostic associations of autoantibodies recognizing adrenergic/muscarinic receptors in patients with heart failure
Source: Cardiovasc Res. 2023 Mar 8;119(8):1690–705. doi: 10.1093/cvr/cvad042 (PMC10325696; doi:10.1093/cvr/cvad042)
Supplement: cvad042_Supplementary_Data [file cvad042_supplementary_data.zip › AABs in HF (Callisto)_Supplementary Methods_3_CVR.docx]

**Supplementary Methods**

**Principles of Immunoluminometric Quantification**

The immunoluminometric quantification of autoantibody (AAB) titers relies on the binding of AABs to recombinant secreted alkaline phosphatase (SEAP)-receptor fusion proteins, followed by isolation of the AAB–fusion protein complexes by protein A/G. Protein complexes are formed, isolated, washed and the enzymatic activity of the reporter moiety of the fusion protein is measured. To that end, serum samples are first incubated with a protein preparation consisting of the recombinant human receptor (β1-, β2-, β3-adrenergic or M2-muscarinic receptor) fused to SEAP as reporter.

**Assay Development for Autoantibody Measurements**

Assay development followed a similar strategy based on fusion proteins with a reporter enzyme, as described previously for other G-protein coupled receptors.^1,2^ The reading frames of full length adrenergic and M2 reporters were each amplified with polymerase chain reactions and cloned in frame to SEAP with the expression plasmid pIRESneo (Clontech, Palo Alto, CA, USA) as backbone. The plasmids were transfected into HEK293 cells, stable cell clones were generated and propagated, and recombinant fusion proteins were produced and prepared as described previously.^3^ A diluted aliquot (50 µl) with defined reporter enzyme activity is added to 5 µl of serum sample per measurement and incubated with the fusion proteins overnight at 4 °C. Complexes of SEAP-AABs were first isolated with protein A slurry (ASKA Biotech GmbH, Berlin, Germany) and subsequently washed and resuspended in enzyme assay buffer. Lastly, reporter enzyme activity was detected as luminescence in a luminometer (Berthold Technologies, GmbH, Bad Wildbach, Germany). Signals were recorded as relative light units (RLU) and expressed as binding index. The binding index is calculated from the measurements in relation to background noise. To this end, the average RLU obtained from the lowest 50% of signals in the same 96-well assay plate is calculated and set as BI = 1.0. Each measured signal is then divided by this average background RLU value to obtain a specific BI per sample (representing a factor of RLU signal above background noise of BI=1). Mathematical outlier criteria are used to finally separate AAB positive from negative samples, and thereby to classify the respective patients as seropositive or seronegative.

A monoclonal antibody to the N-terminal extracellular domain of the β1-adrenergic receptor (anti-B1AR, IMX-MAB4269, ImmunometriX UG) was used as control for assay specificity. During the analyses, the inter-assay coefficient of variation was below 20%, based on the analysis of the positive control samples included into each microtiter assay plate.

**Explanation on Subjects Classified as Having an Intermediate Autoantibody Status**

As described in the main manuscript, patients and controls were classified into seronegative, intermediate, and seropositive. With regard to the existence of an “intermediate” group, it is a consequence of the fact that the noise in a chemiluminescence assay produces a distribution of signals that partly overlap with the signals generated by the presence of low concentrations of receptor specific AABs. By determining cut-off points for defining a negative and positive signal in this manner, it becomes feasible in an explorative analysis of patient samples to identify diagnostic thresholds for clinically relevant AAB concentrations.

**References**

1. Schniewind HA, Sattler LM, Haudum CW, Münzker J, Minich WB, Obermayer-Pietsch B, Schomburg L. Autoimmunity to the Follicle-Stimulating Hormone Receptor (FSHR) and Luteinizing Hormone Receptor (LHR) in Polycystic Ovarian Syndrome. *Int J Mol Sci* 2021;**22**.

2. Sattler LM, Schniewind HA, Minich WB, Haudum CW, Niklowitz P, Münzker J, Kovács GL, Reinehr T, Obermayer-Pietsch B, Schomburg L. Natural autoantibodies to the gonadotropin-releasing hormone receptor in polycystic ovarian syndrome. *PLoS One* 2021;**16**.

3. Sun Q, Mehl S, Renko K, Seemann P, Görlich CL, Hackler J, Minich WB, Kahaly GJ, Schomburg L. Natural Autoimmunity to Selenoprotein P Impairs Selenium Transport in Hashimoto’s Thyroiditis. *Int J Mol Sci* 2021;**22**.
