# Supplementary material for: Clinical and prognostic associations of autoantibodies recognizing adrenergic/muscarinic receptors in patients with heart failure
Source: Cardiovasc Res. 2023 Mar 8;119(8):1690–705. doi: 10.1093/cvr/cvad042 (PMC10325696; doi:10.1093/cvr/cvad042)
Supplement: cvad042_Supplementary_Data [file cvad042_supplementary_data.zip › AABs in HF (Callisto)-BIOSTAT-CHF supp fig_tab.docx]

**Clinical and prognostic associations of autoantibodies recognizing adrenergic/muscarinic receptors in patients with heart failure**

**George Markousis-Mavrogenis**, MD (Hons); **Waldemar B. Minich**, MSc, PhD; **Ali A. Al-Mubarak**, MD; **Stefan D. Anker**, MD, PhD; **John G.F. Cleland**, MD, PhD; **Kenneth Dickstein**, MD, PhD; **Chim C. Lang**, MD; **Leong L. Ng**, MD; **Nilesh J. Samani**, MD; **Faiez Zannad**, MD, PhD, **Marco Metra**, MD, PhD, **Petra Seemann**, MSc, PhD; **Antonia Hoeg**, MSc, PhD; **Patricio Lopez**, MSc, PhD; **The BIOSTAT-CHF Consortium**; **Dirk J. van Veldhuisen**, MD, PhD; **Rudolf A. de Boer**, MD, PhD; **Adriaan A. Voors**, MD, PhD; **Peter van der Meer**, MD, PhD; **Lutz Schomburg*^✝^**, MSc, PhD; **Nils Bomer***, MSc, PhD.

*These authors have contributed equally to the manuscript.

✝Corresponding author

**Short Title**: Autoantibodies in Heart Failure

**Keywords**: beta 1, beta 2, beta 3, M2, immune system, autoimmunity

**Word Count**: 3849 words

**Address for correspondence**: Prof. Lutz Schomburg, Charité – Universitätsmedizin Berlin, 10115 Berlin, Germany. Email address: lutz.schomburg@charite.de

**Supplementary Figures and Tables**

**
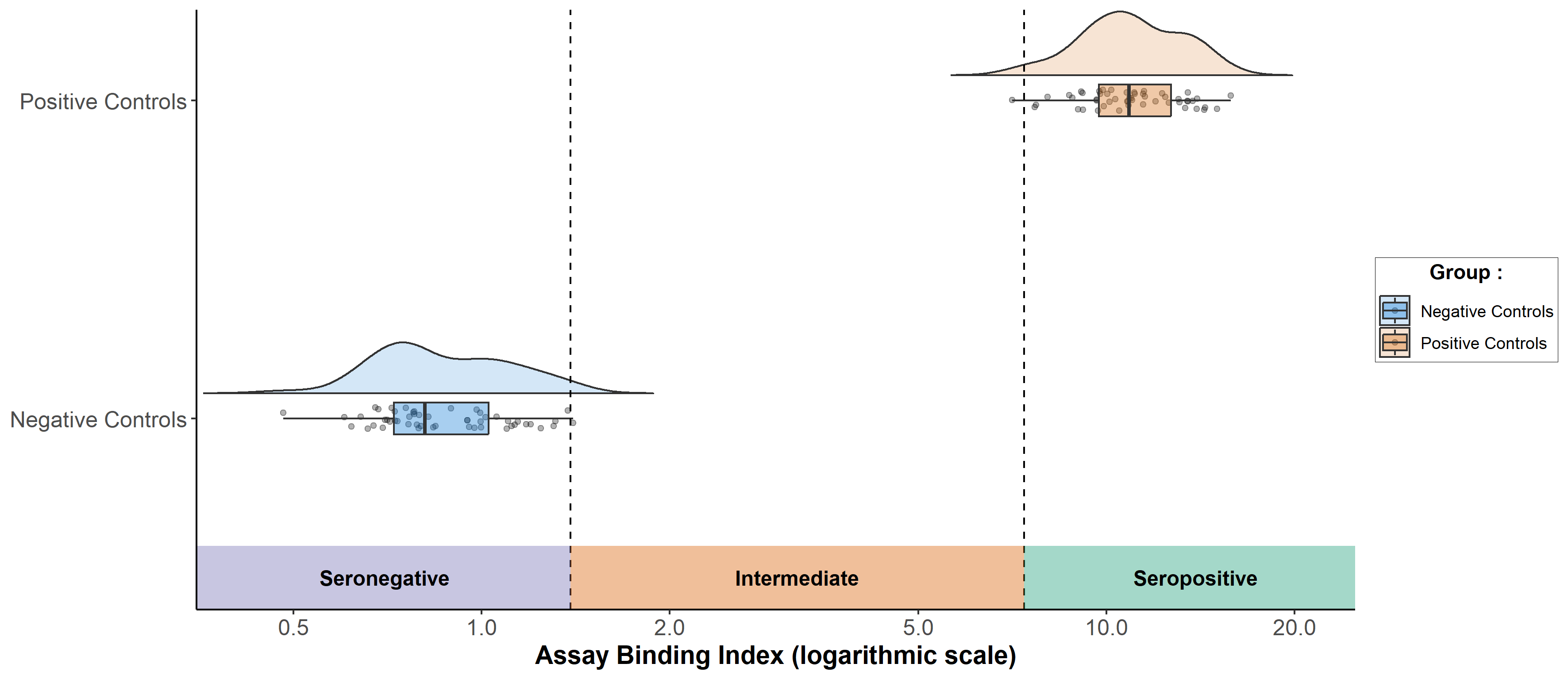
**

**Supplementary Figure 1**. Assay results (binding indices) for the 52 measurements of the negative and positive controls each, that were used to define the cut-off points for seronegative, intermediate, and seropositive classifications, based on the 99th percentile of negative control binding indices and the 1st percentile of positive control binding indices.


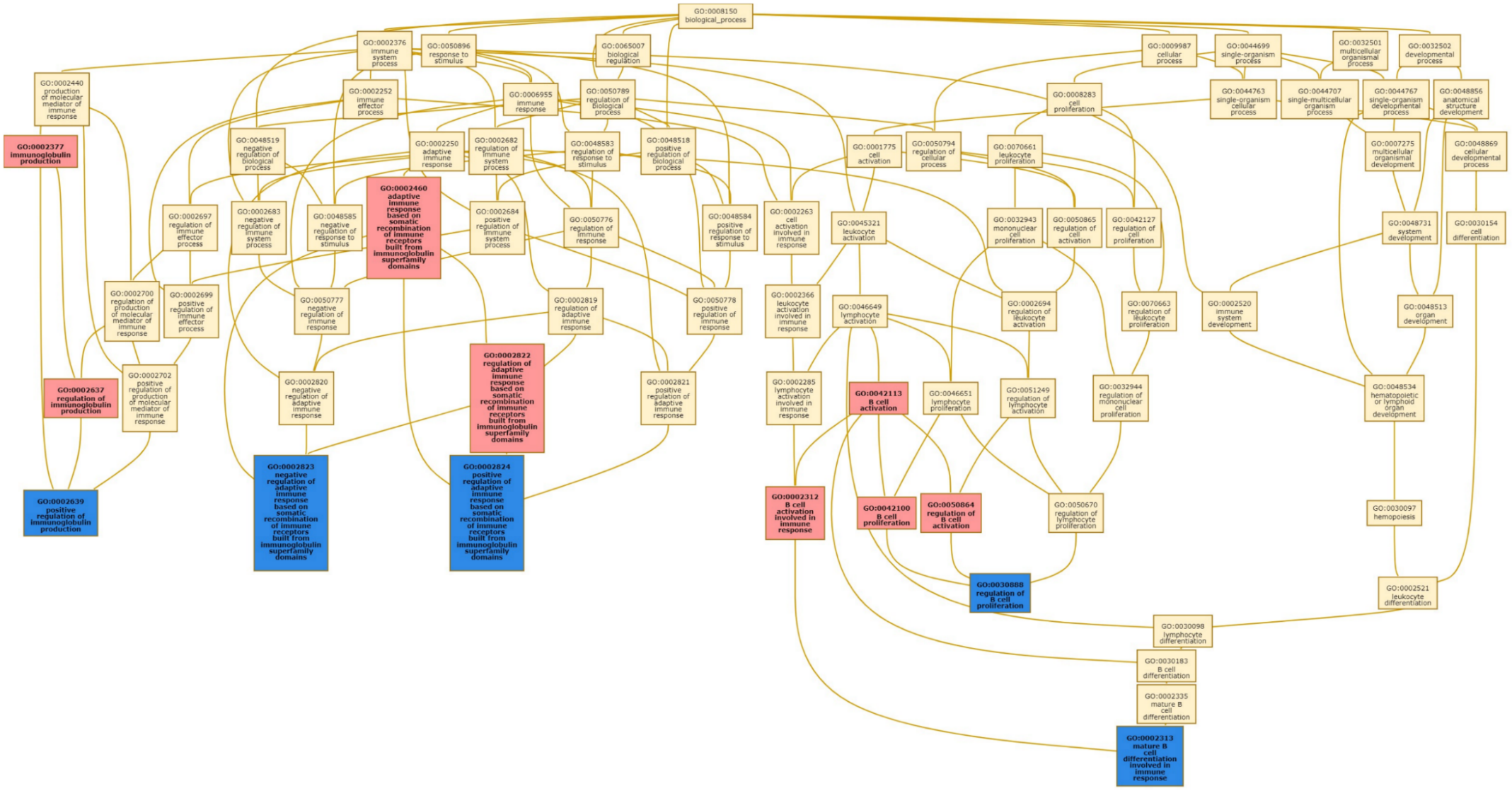
**Supplementary Figure 2**. A high-definition version of the directed acyclic graph shown in **Figure 1A**. The 13 highlighted processes contain the terms “B cell” and/or “immunoglobulin”, and those highlighted in blue specifically are non-redundant children terms that were used for further analyses. GO gene ontology.

**
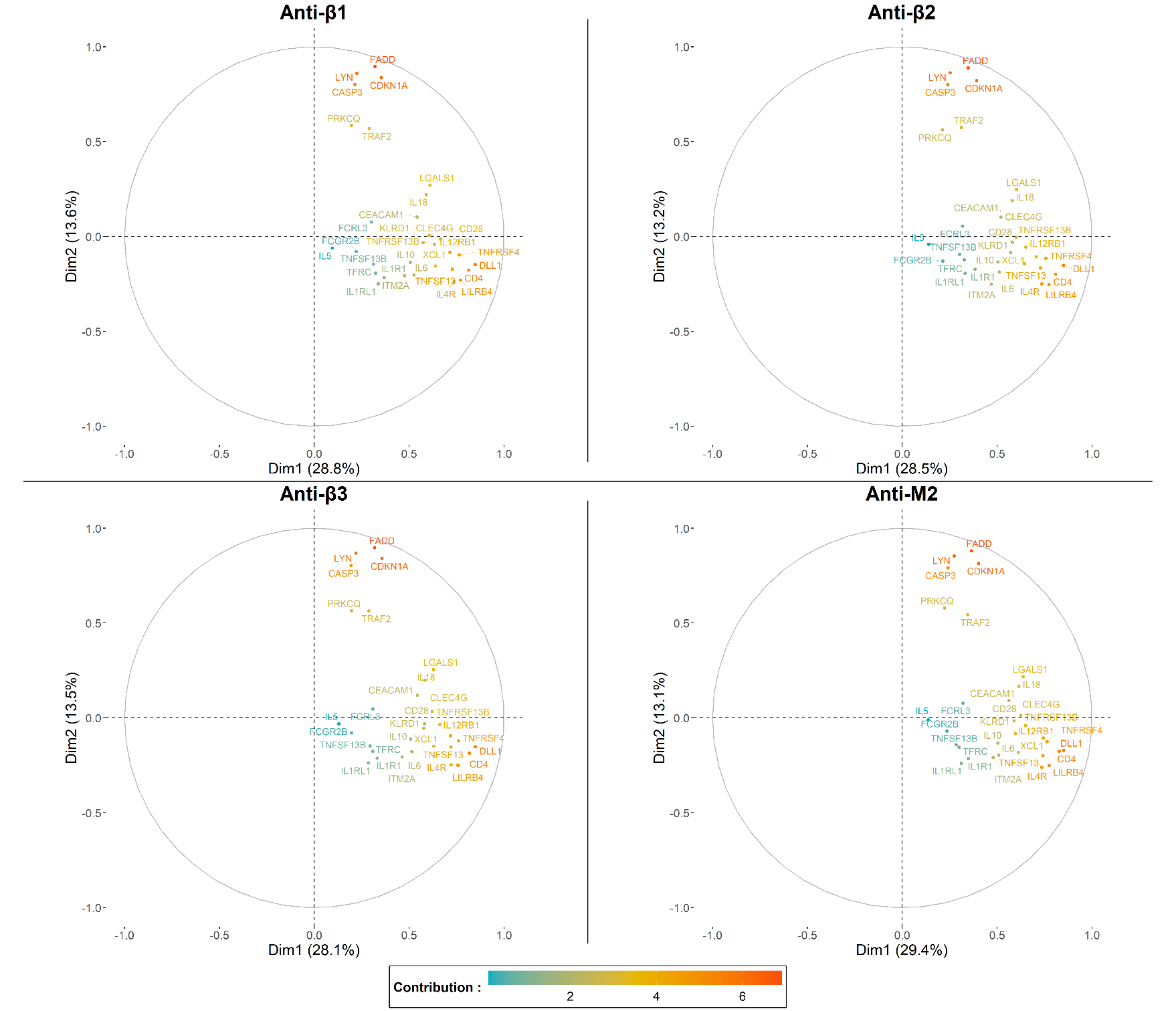
Supplementary Figure 3**. Complete contributions of all examined biomarkers related to B-lymphocyte function to the corresponding principal component analysis of each autoantibody.

| **Supplementary Table 1**. Summary statistics for the assay binding index of all measured autoantibodies for healthy controls and patients with heart failure. | | | | | | | | | | | |
| --- | --- | --- | --- | --- | --- | --- | --- | --- | --- | --- | --- |
| **Group** | **Autoantibody** | **Minimum** | **1^st^ Prct.** | **5^th^ Prct.** | **25^th^ Prct.** | **Median** | **75^th^ Prct.** | **95^th^ Prct.** | **99^th^ Prct.** | **Maximum** | |
| **Healthy Controls (n=299)** | **Anti-β1** | 0.62 | 0.73 | 0.84 | 1.00 | 1.22 | 1.59 | 2.98 | 14.13 | 19.80 | |
|  | **Anti-β2** | 0.47 | 0.64 | 0.75 | 0.97 | 1.24 | 2.01 | 8.27 | 14.44 | 94.60 | |
|  | **Anti-β3** | 0.41 | 0.61 | 0.69 | 0.89 | 1.10 | 1.39 | 2.23 | 6.87 | 37.73 | |
|  | **Anti-M2** | 0.32 | 0.38 | 0.71 | 1.05 | 1.32 | 2.02 | 5.08 | 36.30 | 148.04 | |
| **Patients with Heart Failure (n=2256)** | **Anti-β1** | 0.43 | 0.72 | 0.81 | 1.01 | 1.23 | 1.73 | 7.46 | 27.49 | 120.39 | |
|  | **Anti-β2** | 0.41 | 0.70 | 0.80 | 1.00 | 1.25 | 1.88 | 8.46 | 34.55 | 161.78 | |
|  | **Anti-β3** | 0.44 | 0.73 | 0.83 | 1.01 | 1.19 | 1.46 | 3.30 | 12.67 | 154.00 | |
|  | **Anti-M2** | 0.20 | 0.65 | 0.78 | 1.00 | 1.31 | 2.25 | 12.02 | 46.31 | 170.08 | |
| Prct. Percentile. | | | | | | | | | | |  |

| **Supplementary Table 2**. Summary statistics for the assay binding index of all measured autoantibodies stratified by status (seronegative, intermediate, seropositive) as defined in the methods section. | | | | | | | | | | | | | | |
| --- | --- | --- | --- | --- | --- | --- | --- | --- | --- | --- | --- | --- | --- | --- |
| **Group** | **Autoantibody** | **Status** | **Group Size** | **Minimum** | **1^st^ Prct.** | **5^th^ Prct.** | **25^th^ Prct.** | **Median** | **75^th^ Prct.** | **95^th^ Prct.** | **99^th^ Prct.** | **Maximum** | |  |
| **Healthy Controls (n=299)** | **Anti-β1** | Seronegative  Intermediate  Seropositive | 195 (65.2%)  97 (32.4%)  7 (2.3%) | 0.62  1.39  7.89 | 0.64  1.39  8.14 | 0.80  1.42  9.13 | 0.95  1.56  12.39 | 1.06  1.72  14.09 | 1.20  2.14  16.88 | 1.35  3.24  19.18 | 1.38  4.86  19.68 | 1.39  5.06  19.80 | |  |
|  | **Anti-β2** | Seronegative  Intermediate  Seropositive | 174 (58.2%)  107 (35.8%)  18 (6.0%) | 0.47  1.39  7.63 | 0.58  1.40  7.67 | 0.70  1.42  7.83 | 0.86  1.58  9.05 | 1.00  2.12  12.04 | 1.17  3.30  13.83 | 1.33  6.21  77.74 | 1.37  7.20  91.22 | 1.38  7.31  94.60 | |  |
|  | **Anti-β3** | Seronegative  Intermediate  Seropositive | 223 (74.6%)  73 (24.4%)  3 (1.0%) | 0.41  1.39  8.08 | 0.60  1.39  8.19 | 0.68  1.42  8.64 | 0.86  1.55  10.86 | 1.02  1.78  13.64 | 1.14  2.08  25.68 | 1.31  4.84  35.32 | 1.37  6.46  37.25 | 1.39  6.85  37.73 | |  |
|  | **Anti-M2** | Seronegative  Intermediate  Seropositive | 159 (53.2%)  129 (43.1%)  11 (3.7%) | 0.32  1.39  7.62 | 0.34  1.40  7.81 | 0.54  1.46  8.56 | 0.89  1.64  10.88 | 1.06  2.01  19.20 | 1.21  2.67  39.03 | 1.33  4.91  111.85 | 1.37  5.77  140.80 | 1.38  6.05  148.04 | |  |
| **Patients with Heart Failure (n=2256)** | **Anti-β1** | Seronegative  Intermediate  Seropositive | 1399 (62.0%)  743 (32.9%)  114 (5.1%) | 0.43  1.39  7.46 | 0.69  1.39  7.48 | 0.78  1.42  7.84 | 0.93  1.57  8.99 | 1.05  1.92  11.29 | 1.19  2.74  20.49 | 1.33  5.56  62.06 | 1.37  7.01  111.56 | 1.39  7.30  120.39 | |  |
|  | **Anti-β2** | Seronegative  Intermediate  Seropositive | 1326 (58.8%)  797 (35.3%)  133 (5.9%) | 0.41  1.39  7.43 | 0.66  1.40  7.48 | 0.77  1.43  7.96 | 0.91  1.60  9.53 | 1.04  1.94  13.28 | 1.19  2.86  27.57 | 1.33  5.37  60.69 | 1.38  6.67  127.96 | 1.39  7.36  161.78 | |  |
|  | **Anti-β3** | Seronegative  Intermediate  Seropositive | 1575 (69.8%)  640 (28.4%)  41 (1.8%) | 0.44  1.39  7.44 | 0.71  1.39  7.61 | 0.81  1.41  7.86 | 0.96  1.49  10.10 | 1.08  1.68  13.67 | 1.21  2.14  44.90 | 1.34  4.42  122.58 | 1.38  6.54  145.55 | 1.39  6.93  154.00 | |  |
|  | **Anti-M2** | Seronegative  Intermediate  Seropositive | 1221 (54.1%)  863 (38.3%)  172 (7.6%) | 0.20  1.39  7.49 | 0.63  1.39  7.51 | 0.72  1.43  7.74 | 0.89  1.64  10.79 | 1.02  2.11  15.60 | 1.18  3.23  27.97 | 1.33  5.75  90.28 | 1.37  6.94  142.30 | 1.39  7.37  170.08 | |  |
| Prct. Percentile. | | | | | | | | | | | | |  |  |

| **Supplementary Table 3**. Subgroup analyses of patients with intermediate AAB status using Cox regression for the combined endpoint and all-cause mortality, and competing risks regression for HF-rehospitalization. In cases where univariable findings were significant, multivariable correction for the corresponding risk model for each outcome was carried out. | | | | | | | | | | | |
| --- | --- | --- | --- | --- | --- | --- | --- | --- | --- | --- | --- |
| **AAB** | **Combined Outcome** | | | | | **All-Cause Mortality** | | | | **HF-Rehospitalization** | |
|  | **Univariable** | | | **Multivariable** | | **Univariable** | | **Multivariable** | | **Univariable** | |
|  | **HR (95% CI)** | **p-value** | **HR (95% CI)** | | **p-value** | **HR (95% CI)** | **p-value** | **HR (95% CI)** | **p-value** | **HR (95% CI)** | **p-value** |
| **Anti-β1** | 0.98 (0.89-1.07) | 0.632 | N/A | | | 0.95 (0.84-1.08) | 0.447 | N/A | | 1.03 (0.92-1.15) | 0.630 |
| **Anti-β2** | 0.93 (0.85, 1.01) | 0.119 |  |  |  | 0.83 (0.72-0.95) | **0.008*** | 0.92 (0.80-1.05) | 0.196 | 0.98 (0.88-1.10) | 0.740 |
| **Anti-β3** | 0.82 (0.71-0.94) | **0.006*** | 0.83 (0.72-0.95) | | **0.009*** | 0.85 (0.71-1.01) | 0.063 | N/A | | 0.84 (0.69-1.03) | 0.09 |
| **Anti-M2** | 1.05 (0.98-1.13) | 0.187 | N/A | | | 1.04 (0.95-1.14) | 0.356 |  |  | 1.06 (0.97-1.17) | 0.190 |
| AAB autoantibody; HR hazard ratio; 95% CI 95% confidence interval; *p≤0.05. | | | | | | | | | | | |
