# Supplementary figures and images for: Clinical and prognostic associations of autoantibodies recognizing adrenergic/muscarinic receptors in patients with heart failure
Source: Cardiovasc Res. 2023 Mar 8;119(8):1690–705. doi: 10.1093/cvr/cvad042 (PMC10325696; doi:10.1093/cvr/cvad042)

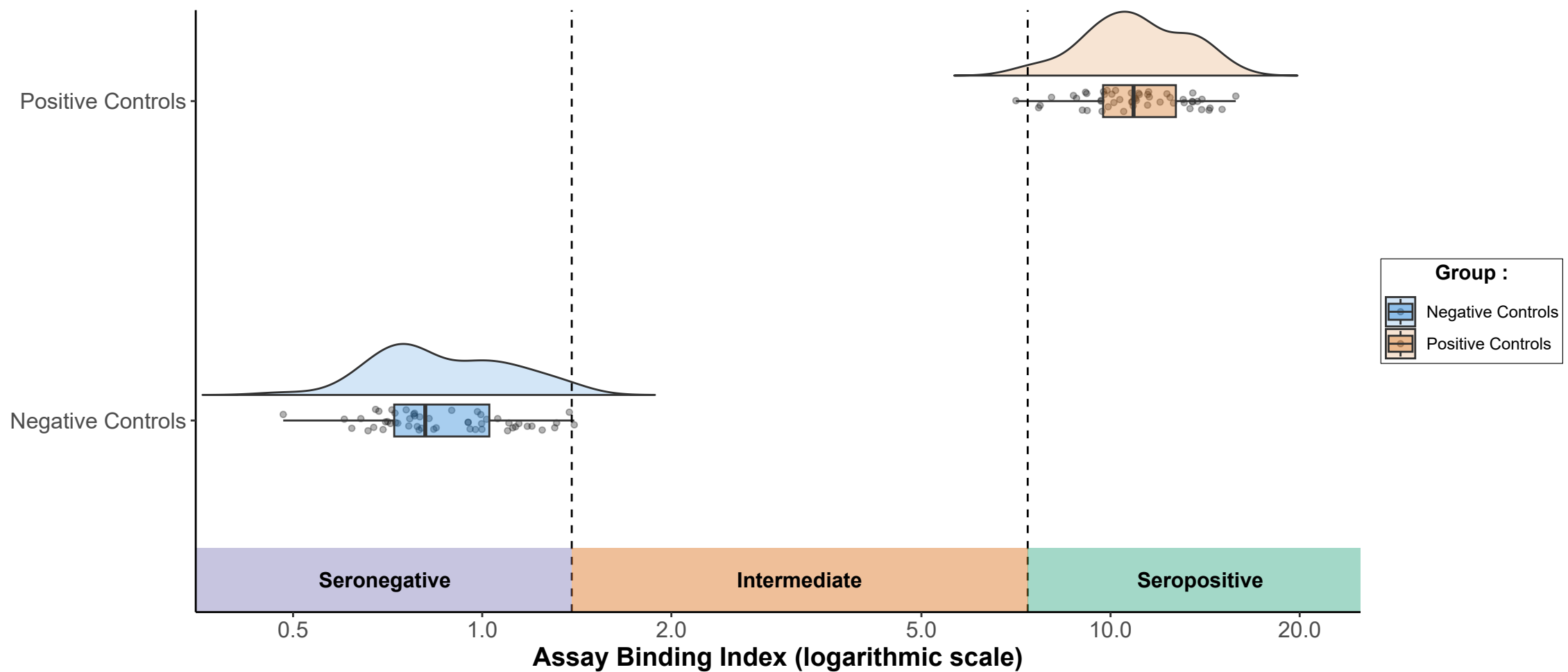

Supplement: cvad042_Supplementary_Data [file cvad042_supplementary_data.zip › Supplementary Figure 1.pdf]

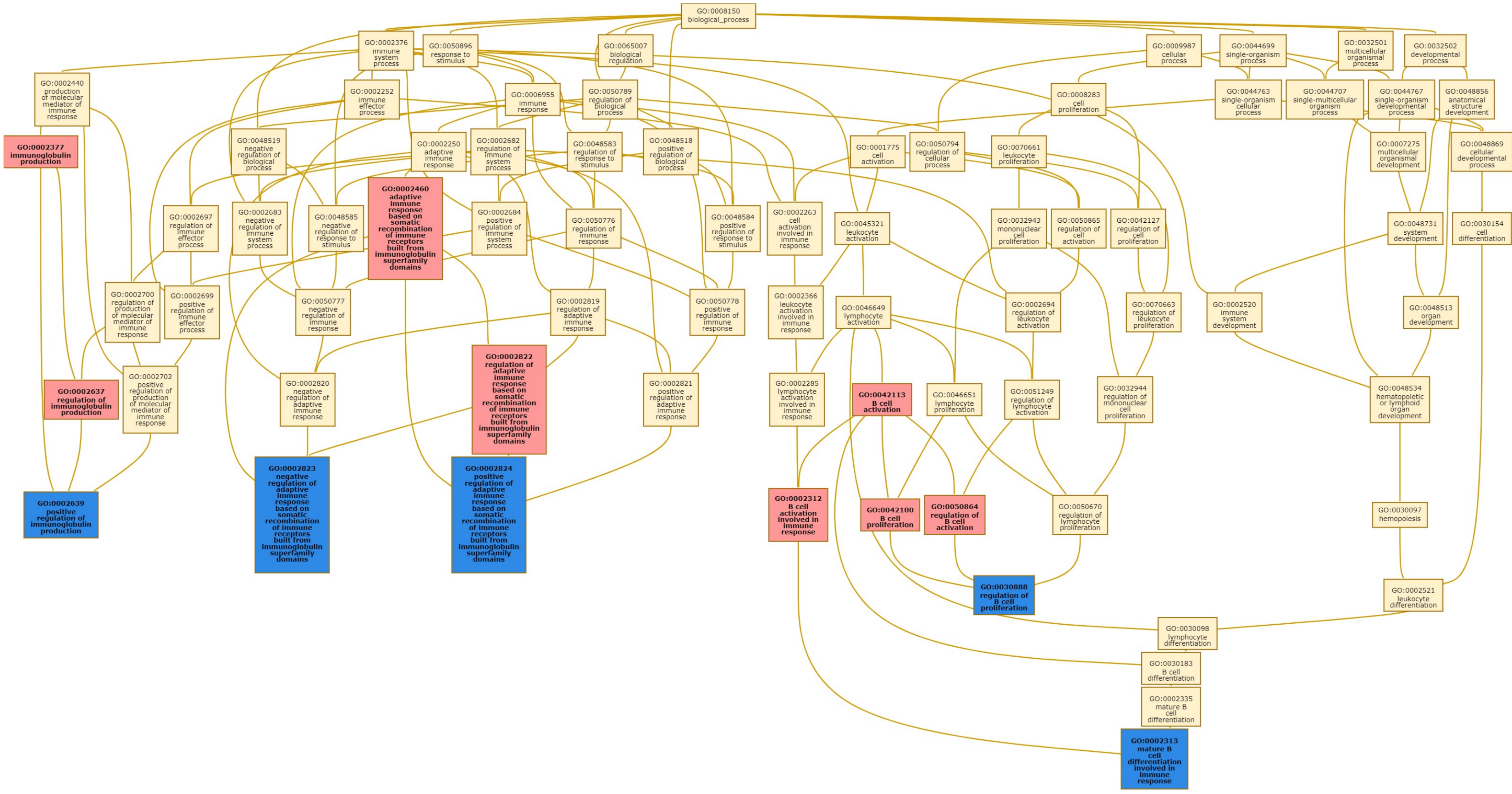

Supplement: cvad042_Supplementary_Data [file cvad042_supplementary_data.zip › Supplementary Figure 2.pdf]

Anti-β1

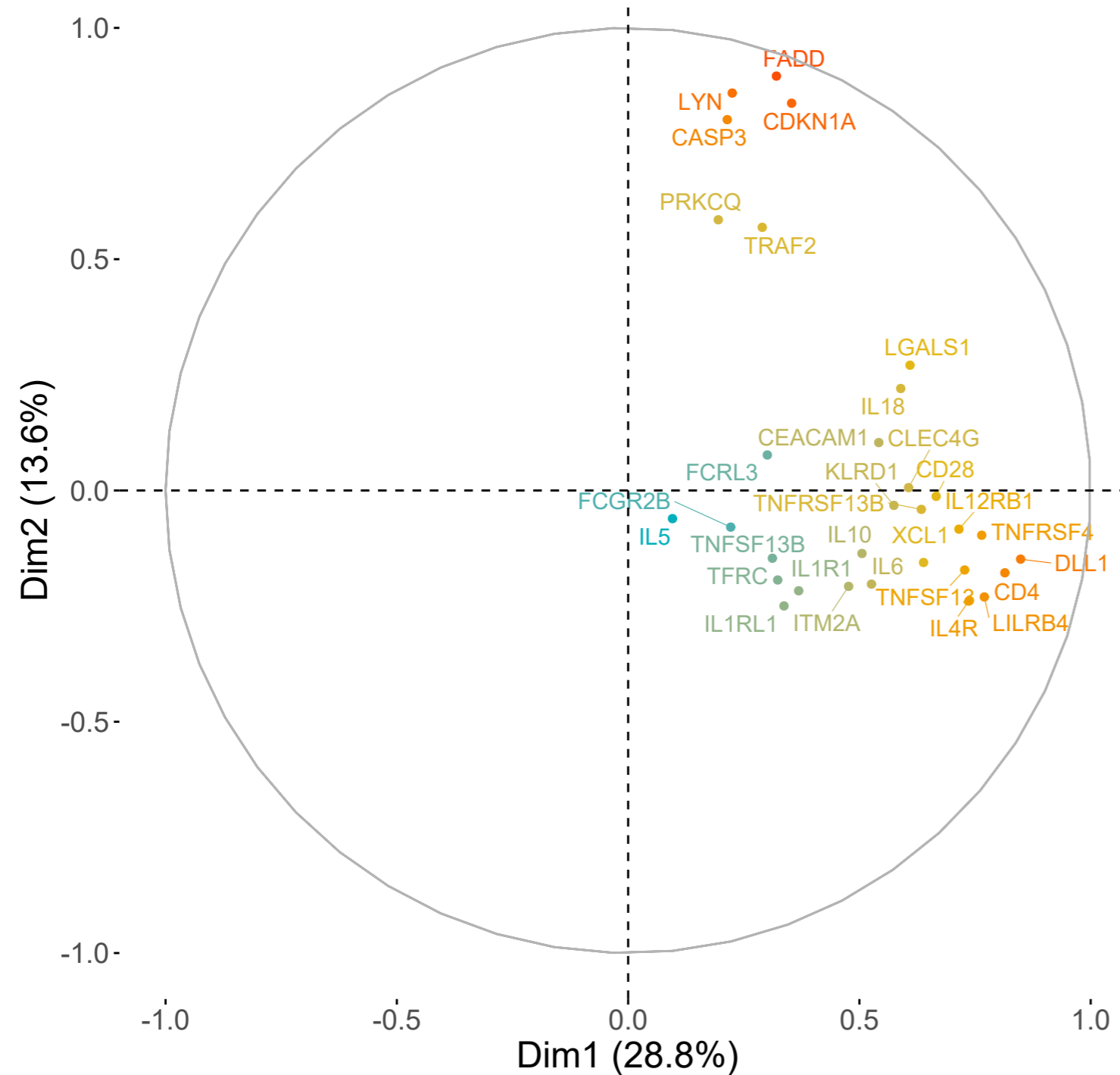

Anti-β2

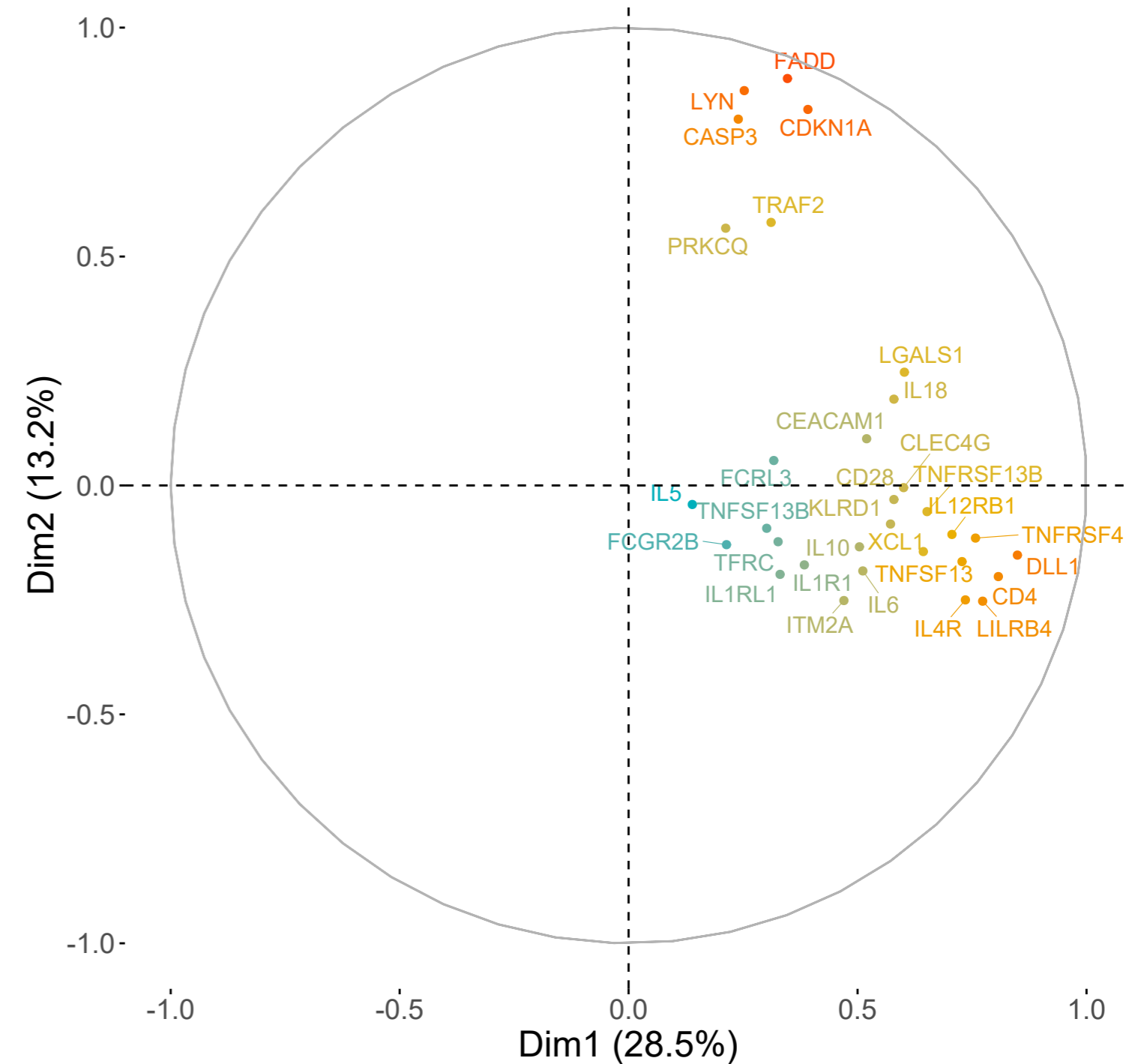

Anti-β3

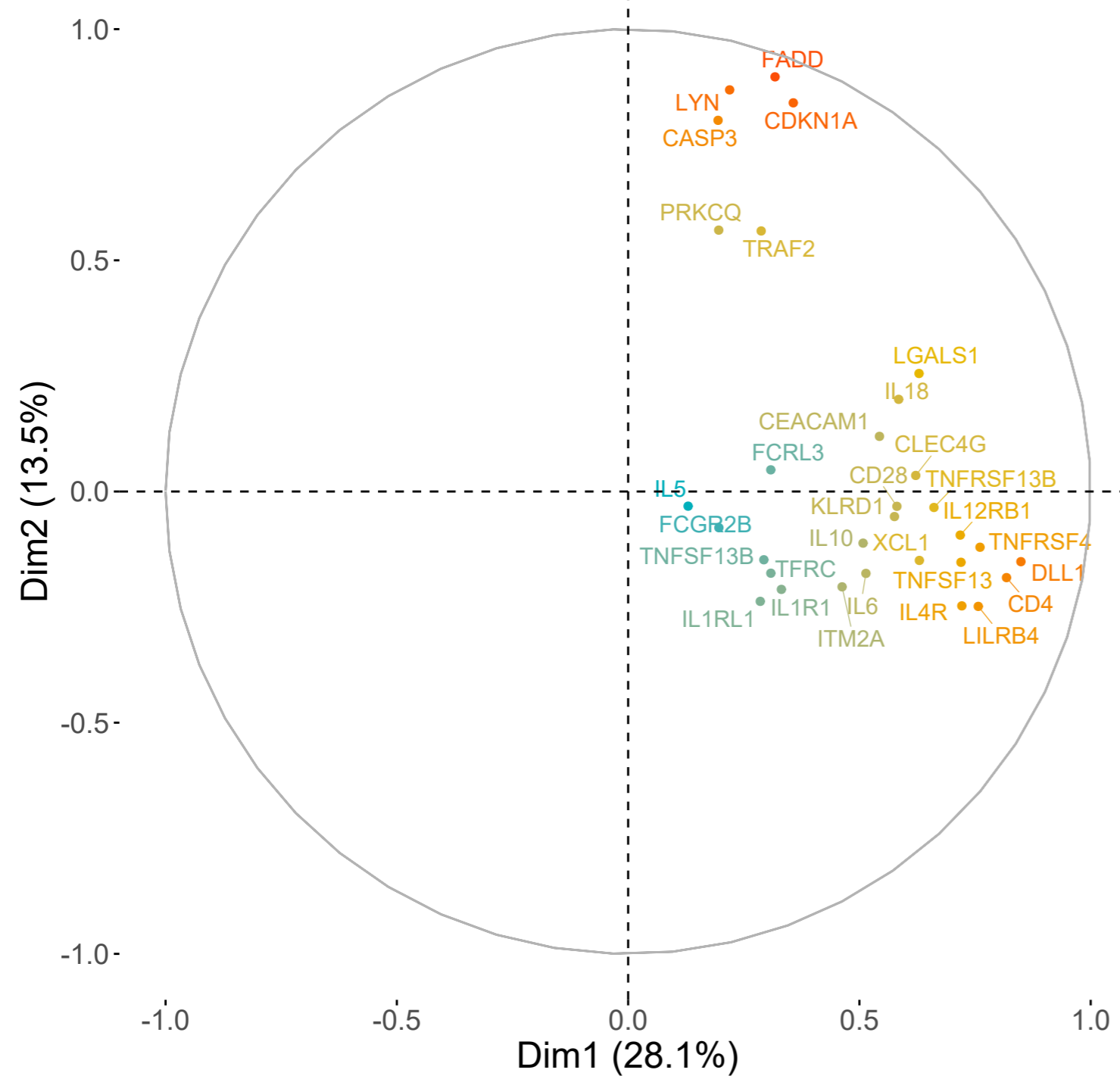

Anti-M2

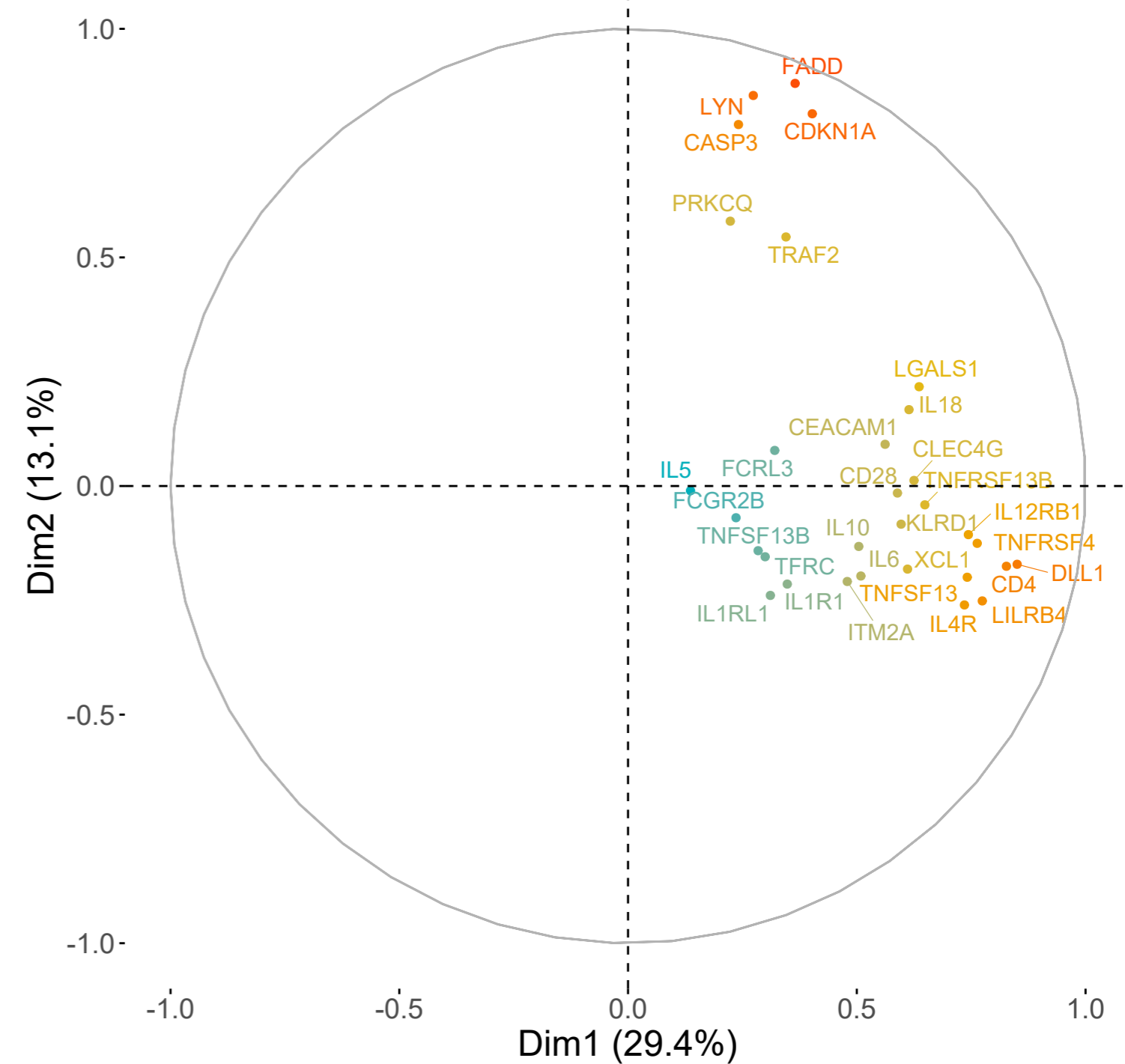

Contribution :

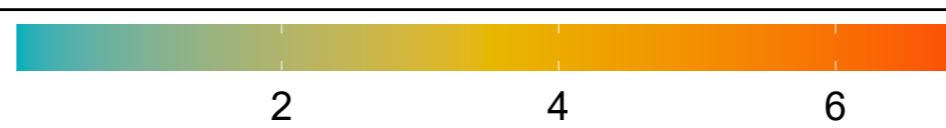

Supplement: cvad042_Supplementary_Data [file cvad042_supplementary_data.zip › Supplementary Figure 3.pdf]
